# Incorporation of a Metal Catalyst for the Ammonia Synthesis in a Ferroelectric Packed-Bed Plasma Reactor: Does It Really Matter?

**DOI:** 10.1021/acssuschemeng.2c05877

**Published:** 2023-02-20

**Authors:** Paula Navascués, Juan Garrido-García, José Cotrino, Agustín R. González-Elipe, Ana Gómez-Ramírez

**Affiliations:** † Laboratory of Nanotechnology on Surfaces and Plasma. Instituto de Ciencia de Materiales de Sevilla (CSIC-Universidad de Sevilla), Avda. Américo Vespucio 49, E-41092 Seville, Spain; ‡ Departamento de Física Atómica, Molecular y Nuclear, Universidad de Sevilla, Avda. Reina Mercedes, E-41012 Seville, Spain

**Keywords:** ammonia synthesis, nonthermal plasmas, plasma-catalysis, packed-bed reactors, ferroelectric barrier discharge, ruthenium catalyst

## Abstract

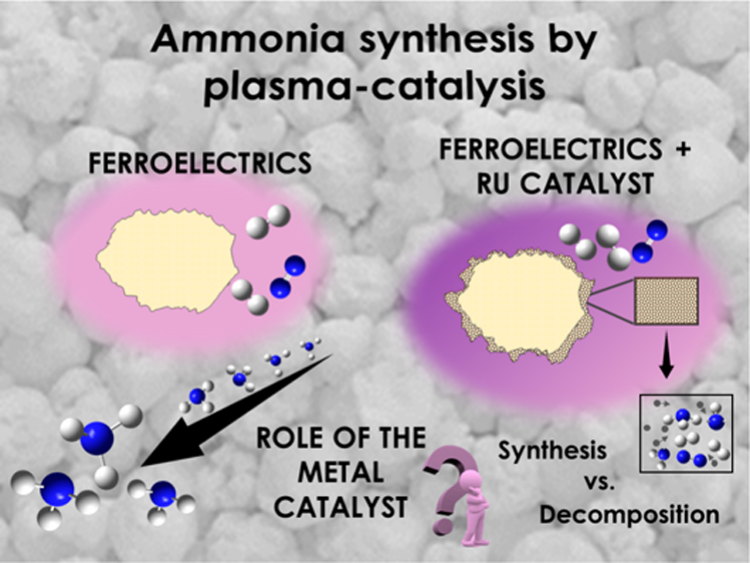

Plasma-catalysis has been proposed as a potential alternative for the synthesis of ammonia. Studies in this area focus on the reaction mechanisms and the apparent synergy existing between processes occurring in the plasma phase and on the surface of the catalytic material. In the present study, we approach this problem using a parallel-plate packed-bed reactor with the gap between the electrodes filled with pellets of lead zirconate titanate (PZT), with this ferroelectric material modified with a coating layer of alumina (i.e., Al_2_O_3_/PZT) and the same alumina layer incorporating ruthenium nanoparticles (i.e., Ru-Al_2_O_3_/PZT). At ambient temperature, the electrical behavior of the ferroelectric packed-bed reactor differed for these three types of barriers, with the plasma current reaching a maximum when using Ru-Al_2_O_3_/PZT pellets. A systematic analysis of the reaction yield and energy efficiency for the ammonia synthesis reaction, at ambient temperature and at 190 °C and various electrical operating conditions, has demonstrated that the yield and the energy efficiency for the ammonia synthesis do not significantly improve when including ruthenium particles, even at temperatures at which an incipient catalytic activity could be inferred. Besides disregarding a net plasma-catalysis effect, reaction results highlight the positive role of the ferroelectric PZT as moderator of the discharge, that of Ru particles as plasma hot points, and that of the Al_2_O_3_ coating as a plasma cooling dielectric layer.

## Abstract 2

Incorporation of metal catalysts in packed-bed plasma reactors for the ammonia synthesis does not contribute to making this technology competitive.

## Introduction

Ammonia is a strategic compound because of its central role to produce nitrogen fertilizers, which are indispensable to increase the crops productivity in order to feed the growing world population. It is also deemed a suitable and safe hydrogen vector and therefore a strategic compound for the energy ecosystem transformation. Currently, ammonia is obtained through the catalytically driven Haber–Bosch process, which proceeds at high pressures and temperatures with yields around 15–20%.^1^ Today, ammonia production exceeds 150 Mt per year; it is responsible for 1.2% of the worldwide greenhouse gases emission^2−4^ and consumes ca. 2% of the world energy supply.^5^ In the race to make the chemical industry more sustainable (i.e., free from CO_2_ emissions) and compatible with intermittent electricity sources, the search for alternative procedures to produce ammonia has become a key topical issue. In this context, plasma technology has emerged as an attractive candidate for the ammonia synthesis because it offers mild operation conditions (ambient temperature and atmospheric pressure), easiness to scale-up the reactors, and the possibility to operate in a distributed way directly connected to the grid or to decentralized small-scale renewable power plants.^6^

Among the different plasma technologies used for the NH_3_ synthesis (e.g., gliding arc discharges,^7^ plasma jets,^8^ or RF discharges^9^), packed-bed plasma processes are being widely investigated since the reactors permit the direct incorporation of a catalyst in the barrier, a combination that has been claimed to render superior process performance.^10^ Nevertheless, the reason(s) for this apparent synergy is still a debatable trending question^11^ and an appealing challenge to the plasma-catalysis community. For example, although the kind of metal used as catalyst,^12^ the type of support,^13^ its dielectric constant,^14^ mesoporosity,^5^ specific surface area,^15^ or even its size^16^ are known to influence the plasma–catalyst interaction, and hence the reaction kinetics and yields, there is not yet a clear understanding of the effect of each particular parameter, thus hampering the systematic design of more efficient plasma–catalyst systems.

Many authors have worked in the selection of appropriated catalysts for the plasma-assisted synthesis of NH_3_. In this regard, Akay and Zhang obtained a 12% nitrogen conversion using a porous Ni-based catalyst in a discharge moderated by glass and BaTiO_3_ pellets.^17^ Tu and collaborators have recently studied the effect of incorporating a metal catalyst into BaTiO_3_ pellets, obtaining energy efficiency values higher than 2 g of NH_3_/kWh when using nickel particles.^12^ Carreon and co-workers have reported that perovskites with electronegative alkaline-earth cations (e.g., MgTiO_3_) contribute to weaken the nitrogen bond (N≡N) and thereby favor the ammonia synthesis reaction.^14^ These authors have also demonstrated that mesoporous materials such as zeolites seem to favor the ammonia synthesis.^18,19^ Similar results have been obtained by Wang et al. using a Ni catalyst supported on a mesoporous MCM-41 support^5^ and by Rouwenhorst et al.,^20^ using zeolite 4A as adsorbent. Proposed explanations for the positive role of nanoporous materials as catalysts assume that the adsorption of the formed ammonia molecules in porous structures prevents their decomposition (i.e., the reverse reaction, 2NH_3_ → 3H_2_ + N_2_ (1), which can be promoted by plasma electrons). Recently, we have also demonstrated that inefficient reactions involving hydrogen exchange processes (e.g., NH_3_ + H_2_ → NH_2_H + H_2_ (2)) can also take place in packed-bed reactors, contributing to decrease the energy efficiency of the synthesis reaction.^21^

However, despite the significant contributions to this topic, there are not clear criteria for the choice of suitable catalysts to work under plasma conditions, particularly because plasma catalysts might be different materials than those used for conventional thermal catalytic reactions. In this regard, Bogaerts and co-workers have combined a catalytic microkinetic model with a plasma chemical kinetics description^22^ and concluded that the NH_3_ turnover frequency (TOF) does not depend on the type of catalytic material in plasmas with a high concentration of radical species. This conclusion differs from that in other studies proposing that the TOF depends on the type of catalyst.^15,23,24^ In this line, an experimental work by Gorbanev et al., using Al_2_O_3_-supported Fe, Ru, Co, and Cu catalysts, showed that the reaction yield of ammonia, although always lower than 1%, was up to four time higher when comparing efficiencies for metal catalyst and those obtained with bear Al_2_O_3_ beads as barrier.^25^ Similarly, Gorky et al. found an increase in efficiency upon addition of Ni particles to a packed-bed reactor filled with SiO_2_ pellets.^16^ However, the meager reaction yields around 0.5% reported in this latter study makes the evidence inconclusive owing to this very low ammonia yield.

This controversial scenario in relation with the role of metal particles in the plasma-driven ammonia synthesis reactions rises critical questions about the possible links between surface and plasma mechanisms and the extent by which metals may affect the plasma properties and behavior.^26−28^ In other words, although adding a metal catalyst to the packed-material could promote catalytic-driven processes, this might also modify the electric field in the immediacy of the metal nanoparticles, alter the characteristics of the discharge, and likely affect the reaction mechanisms in the plasma phase. A first objective of the present work, beyond the existence of possible catalytic effects, is to determine the occurrence of plasma effects induced by the presence of metal nanoparticles in the packed bed and how these effects may affect the efficiency of the ammonia synthesis process. A second related objective is the verification of possible modifications in the moderator role of a ferroelectric barrier formed by packed pellets of lead zirconate titanate (PZT), a high-dielectric-constant material with a high Curie temperature that in previous works has demonstrated a high efficiency for ammonia synthesis^21,29,31^ and other plasma-driven reactions.^32−34^

To discuss the possible effects of the incorporation of metal particles on the performance of the ferroelectric reactor, as well as to address other relevant mechanistic aspects of the synthesis process, particularly the possible existence of catalytic-driven mechanisms, ammonia reaction yield and energy efficiency of the plasma reaction are taken as relevant magnitudes for analysis. With this purpose, we study the effect of incorporating a ruthenium (Ru) metal catalyst, one of the most widely used metal catalyst for the thermal and plasma-catalytic synthesis of ammonia,^13,22,23,25,35−37^ into the ferroelectric barrier. This study has been complemented with the optical emission spectroscopy (OES) analysis of the plasma discharge. A comparative analysis has been also carried out using three configurations of the barrier: (i) normal PZT pellets (designed as PZT configuration); (ii) PZT pellets covered by an Al_2_O_3_ coating (Al_2_O_3_/PZT configuration), and (iii) PZT pellets with the alumina coating containing Ru nanoparticles (Ru-Al_2_O_3_/PZT configuration). The results obtained demonstrate that the incorporation of ruthenium nanoparticles does not provide a significant enhancement (or this is negligible) either in reaction yield or in energy efficiency with respect to the PZT barrier, even working at temperatures as high as 190 °C, known to define a threshold for plasma-catalysis ammonia synthesis using ruthenium as catalyst.^37^ A critical assessment of the reaction mechanisms supports that, although ruthenium may contribute to produce more intense plasmas by modifying the electric properties of the discharge, it can be also detrimental for the ammonia synthesis through the promotion of undesired reactions (i.e., the aforementioned ammonia decomposition or hydrogen exchange processes). All these processes seem to hide possible catalytic effects induced by the ruthenium nanoparticles. It is also concluded that the synergy found when incorporating ruthenium to the ferroelectric barrier reactor could be also obtained when using any kind of metal, instead of the high-cost catalyst usually employed for thermal catalysis.

## Experimental Section

### Experimental System

The packed-bed reactor used in this work has been described in previous publications, and readers are addressed to these articles and to the Supporting Information for a complete description of the experimental setup.^21,29,31^ The reactor consists of two stainless steel electrodes of 75 mm diameter separated by a gap of 5 mm where pellets of the ferroelectric PZT are placed. PZT pellets (with a mean diameter in the range of 0.5–2 mm) were prepared in our laboratory upon the high-temperature sintering of powders of this material supplied by APC International Ltd. (Pennsylvania, US), as described in a previous article.^32^ PZT was chosen as ferroelectric barrier material because it has demonstrated a better performance for the ammonia synthesis than the more widely utilized BaTiO_3_ ferroelectric.^29^ Moreover, Curie temperatures of 332 °C for the former vs 120 °C for the latter ensure that PZT ferroelectric properties are preserved at high operating temperatures.^38^ The bottom electrode was grounded, and the upper one was connected to a high voltage power amplifier (Trek Inc., Model PD05034), coupled to an AC function generator (Stanford Research Systems, Model DS345). To electrically characterize the discharge, we used an oscilloscope (Agilent Tech., Model DSO-X 3924A) directly connected to a high voltage probe to measure the applied voltage. To collect the transferred charge or the current we used a capacitor (2.51 μF) or a resistance (223 Ω) in series with the plasma reactor when the experiments were carried out at ambient or high temperature, respectively. When the capacitor was used, a current monitor (Pearson, Model 6585) was ground connected to measure the current. The area of the Lissajous curves (plot of transferred charge vs applied voltage) was taken as a measurement of the average consumed power in each experiment. Two series of experiments were performed at ambient temperature: first, we varied the applied voltage amplitude between 1.75 and 3 kV at a fixed frequency of 5 kHz; second, we varied the frequency between 1 and 5 kHz at a fixed voltage of 2.5 kV. This latter procedure induces a progressive change in the plasma current and was chosen as a method to vary systematically the plasma power without modifying the electric field distribution in the packed-bed reactor.

All the experiments have been carried out at atmospheric pressure. Some experiments were performed at ambient temperature (a small drift up to 40 °C under steady state conditions could be detected, an issue that has been considered for energy efficiency calculations) and others at 190 °C, as determined by a thermocouple placed at the external walls of the reactor. To achieve these high-temperature conditions, the stainless-steel reactor was wrapped with heating ribbons activated by a temperature heating control device. At ambient temperature, the frequency was varied between 1 and 5 kHz, while for the 190 °C experiments it was varied between 1 and 3 kHz (keeping constant the voltage at 2.5 kV) because of experimental limitations: at high temperatures, the appearance of sparks and short-circuits affected the stability of the plasma at frequencies higher than 3 kHz.

As inlet gases, we used nitrogen and hydrogen (Air–Liquid,,Alphagaz). A total flow rate of 23 sccm and a N_2_/H_2_ ratio of 1:3 was kept constant during all the experiments. To ensure that the residence time of the reactant gases in the reactor was similar when varying the temperature, we adjusted the gas flow considering the expected volume expansion due to heating inside the reactor. N_2_ and H_2_ gas flows were set at 5.75 and 17.25 sccm at ambient temperature and at 3.7 and 11.1 sccm at 190 °C, respectively.

A quadrupole mass spectrometer (Pfeiffer Vacuum, QMG 220 Prisma Plus) was used to analyze the reaction products. The reaction yield, which accounts for the amount of nitrogen transformed into ammonia, was defined as follows:^21,29,31^
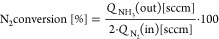
E1where *Q*_NH_3__ (out) and *Q*_N_2__ (in) are the flow rates of the produced ammonia and the nitrogen feeding the reactor, respectively. The energy efficiency of the synthesis process is defined as the ratio between the amount of produced ammonia and the consumed energy:^21,29,31^
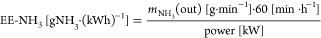
E2where *m*_NH_3__ (out) refers to the mass (in grams per minute) of produced ammonia. This evaluation of NH_3_ mass from the outlet flow takes into account the average temperature of the reactor during each experiment. We should indicate that temperature was measured at the reactor walls and that values of this parameter may be slightly higher in the interior of the reactor.

Optical emission spectra (OES) were recorded with a monochromator (Horiba Ltd., Jobin Yvon FHR640) with a resolution of 0.2 nm. The light emitted by the plasma was collected by an optical fiber feedthrough situated at the lateral wall of the reactor, pointing to the interelectrode space (see details of the diagnosis in the Supporting Information).

### Preparation and Characterization of Coated PZT Pellets

In this study, we have investigated the effects of combining high-dielectric constant ferroelectric pellets (PZT) with a dielectric Al_2_O_3_ coating loaded with metal nanoparticles. Al_2_O_3_ powder was used as support for the metal catalyst due to its high surface area, which allows for a good dispersion of the Ru nanoparticles. From an electrical point of view, it is noteworthy that its dielectric constant—around 10—is much lower than that of the PZT—around 1900 at ambient temperature.

A Ru-Al_2_O_3_ catalyst powder prepared by wet impregnation was incorporated onto the PZT pellets. For comparative purposes, the PZT pellets were also covered with just the Al_2_O_3_ powder. To prepare the Al_2_O_3_/PZT pellets, the Al_2_O_3_ powder (Sigma-Aldrich, γ-Al_2_O_3_ with a BET surface area of 120 m^2^/g) was water impregnated (using an incipient wetting impregnation technique), and the resulted slurry was used to cover the PZT pellets. For the Al_2_O_3_-supported Ru catalyst, the coating process was done using a solution of ruthenium(III) chloride hydrate (Sigma-Aldrich) instead of water. Approximately 5 mL of a ruthenium chloride solution (63 mg/mL) was needed to incorporate a 2 wt % Ru loading into 7 g of Al_2_O_3_ powder. After covering the pellets with the corresponding slurry, they were kept at ambient temperature during 12 h. Then, following the procedure described by Patil,^39^ the pellets were dried in air at 120 °C for 4 h, reaching this temperature with a heating ramp of 1 °C/min to evaporate the water in a smooth way. Finally, the resulting pellets were calcined in air at 450 °C for 3 h, applying a ramp rate of 2 °C/min. After this calcination treatment, ruthenium should be in an oxidized form, but it becomes reduced to metal nanoparticles upon exposure in the reactor to the reducing N_2_+H_2_ plasma. This chemical transformation should be quite fast and occur during the first few seconds of reactor operation.

The coated pellets and the Al_2_O_3_ and Ru-Al_2_O_3_ powders were characterized by means of scanning electron microscopy (SEM) using a S4800 field emission microscope (Hitachi High-Tech Corporation) equipped with an energy dispersive X-ray analyzer at 30 kV (EDX, Bruker-X Flash-4010). The Al_2_O_3_ and Ru-Al_2_O_3_ powders were also characterized by means of transmission electron microscopy (TEM) using a JEOL 2100Plus microscope operated at 200 kV. Surface characteristics of coated pellets and powder samples were determined by X-ray photoelectron spectroscopy (XPS) using a PHOIBOS 100 (Specs) spectrometer operating at normal incidence. The Kα line of aluminum was utilized to collect the spectra. The binding energy (BE) scale was referenced to the C1s line of spurious carbon taken at 284.6 eV. The BET surface areas of the PZT, Al_2_O_3_/PZT, and Ru-Al_2_O_3_/PZT pellets were measured by means of a TriStar II 3020 analyzer (Micromeritics Instruments Corporation). The surface areas of the Al_2_O_3_ and Ru-Al_2_O_3_ powders were also determined. Prior to this analysis, all the samples were outgassed at 150 °C for 2 h.

### Packed-Bed Barrier Configurations

**Figure 1:**
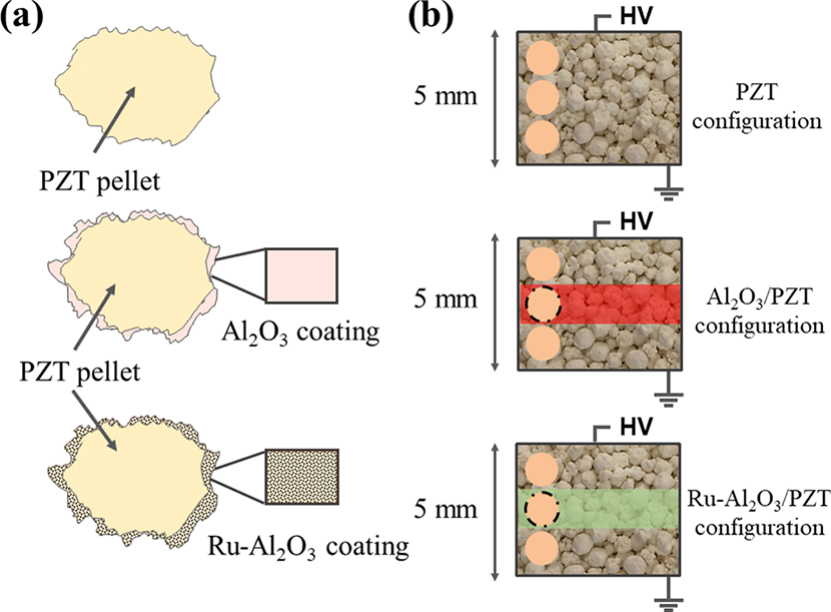
(a) Schematic of PZT, Al_2_O_3_/PZT, and Ru-Al_2_O_3_/PZT pellets. (b) Arrangements of the sets of pellets for the used barrier configurations. Ground and HV (high voltage) symbols refer to the electrical connections of bottom and top electrodes.

The Al_2_O_3_/PZT and Ru-Al_2_O_3_/PZT pellets were introduced in the packed-bed as a compact layer in the middle of the barrier, sandwiched by two layers of PZT pellets. This configuration avoids possible short-circuits due to local discharges and electrical contact between Ru particles and the metal electrodes. The central layer of coated pellets had a volume of 11.5 cm^3^, while the top and bottom layers of PZT pellets occupy a total volume of 27 cm^3^. The sketches in Figure 1 show schematically the differences between the three kinds of pellets, as well as between the barrier architectures of the packed-bed reactor for each configuration.

**Figure 2:**
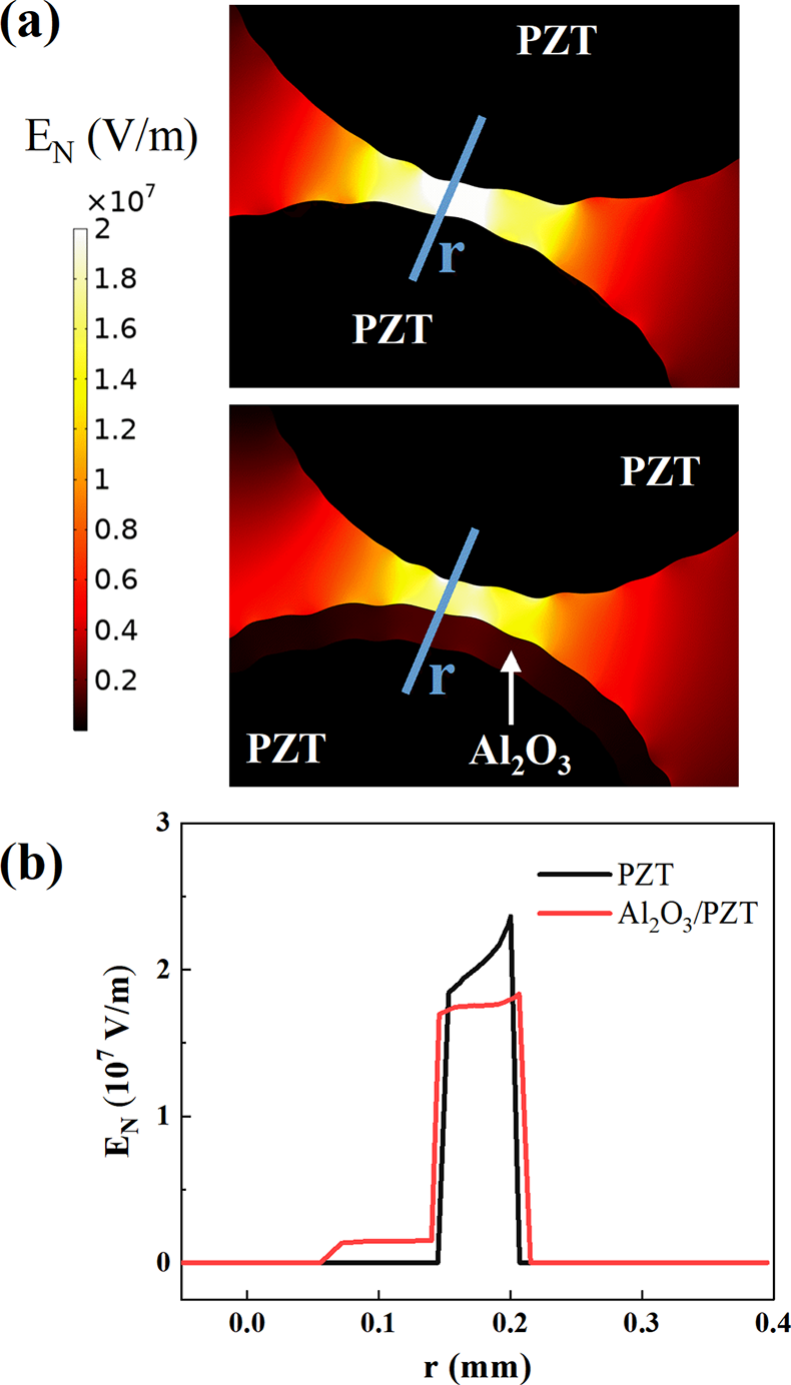
Analysis of the electric field distribution in the packed-bed barrier by means of Comsol Multiphysics simulations.^40^ (a) Distribution of the electric field between two equally separated pellets of PZT (top) and a PZT and a Al_2_O_3_/PZT pellet (bottom). The “r” line indicates the straight line selected to evaluate the electric field. (b) Electric field distribution along the line “r” for the PZT and Al_2_O_3_/PZT configurations.

The effect of covering the PZT pellets with an alumina coating has been analyzed with the AC/DC module of Comsol Multiphysics.^40^ Simulations have been carried out for an interelectrode distance of 3.695 mm, assuming that the PZT pellets are irregular and have a mean radius of 0.6 mm. To disregard any electric field variation due to differences in the pellet surface geometry, the external shape profile of the Al_2_O_3_/PZT is taken identical with that of the PZT pellets but incorporating an irregular alumina coating with a mean thickness of 0.05 mm (see the Supporting Information for a more detailed description of the simulation procedure). The applied voltage between the electrodes was 2.5 kV at a frequency of 5 kHz. An extremely fine mesh was used for the calculations, rendering a computing time of approximately 90 s. The color maps in Figure 2a (top) depict the electrical field distribution between two PZT pellets (i.e., PZT configuration) and (bottom) between adjacent PZT and Al_2_O_3_/PZT pellets (note that in the Al_2_O_3_/PZT configuration only the middle layer in the packed-bed is filled with coated pellets). These color maps evidence that, for similar topographies and distances, the electric field in the interpellet space is higher between two PZT pellets than between a PZT and an adjacent Al_2_O_3_/PZT pellet, as revealed by the evaluation of the electric field along the line “r” connecting two pellets (see Figure 2b).

The differences in electric field distribution in the interpellet space for the PZT and PZT-Al_2_O_3_/PZT configurations respond to the distinct dielectric constants of the ferroelectric PZT and dielectric Al_2_O_3_ materials.^38^ Interestingly, the electric field intensity appears enhanced at locations where either the Al_2_O_3_ or the PZT surfaces present irregularities. The influence of asperities and irregularities is usually overlooked for similar simulations in the literature, where perfectly spherical interelectrode pellets are usually considered for the calculations.^41,42^ These simulations of electrical field distribution forecast that the lower electric field in the interpellet space for the Al_2_O_3_/PZT configuration may affect the electrical behavior of the reactor. In concrete, according to these results, this configuration is expected to *cool down* the plasma, reducing both the electron temperature and density.

## Results and Discussion

### Characterization of Al_2_O_3_/PZT and Ru-Al_2_O_3_/PZT Pellets

**Figure 3:**
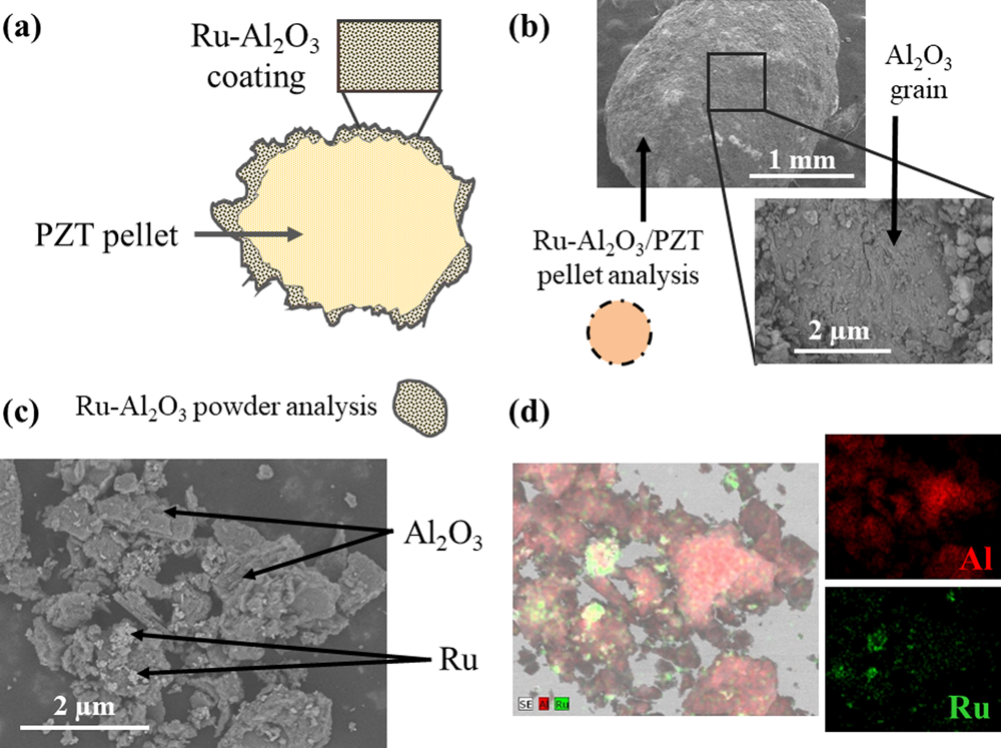
(a) Sketch and (b) SEM micrographs of a Ru-Al_2_O_3_ coated PZT pellet. The dots in sketch (a) refer to Ru aggregates and the fact that it is distributed in the interior of the alumina coating layer. (c) SEM micrograph and (d) EDX analysis in the form of Al (red) and Ru (green) maps of the Al_2_O_3_-supported Ru catalyst powder.

The morphological characteristics of the Ru-Al_2_O_3_/PZT pellets are shown in Figure 3, displaying a series of SEM micrographs and EDX maps of a pellet and its coating. Considering the amount of material used during the wet impregnation process, we can estimate a coating thickness of approximately 0.05 mm, in case it were homogeneous. This corresponds to a 21% of the total pellets volume. A cross section schematic drawing of a Ru-Al_2_O_3_/PZT pellet is shown in Figure 3a. This scheme shows that the coating is irregular and that the Ru nanoparticles are not only located at the external surfaces of the agglomerated Al_2_O_3_ powder but also embedded in pores and between alumina particles in the interior of the irregular alumina coating layer. The micrograph in Figure 3b shows that the coating is not entirely conformal and that the pellet may expose areas of uncovered PZT. A similar topology was found for the Al_2_O_3_/PZT pellets. Additional evidence of this topology is gained by XPS analysis of the surface state of pellets. Data in the Supporting Information show that alumina is the majority surface component, although a non-negligible contribution of PZT (i.e., signals due to Pb, Ti, and Zr) is also detected, indicating that the pellet surface is not fully covered by the catalyst. Meanwhile, the EDX/SEM analysis of the Ru-Al_2_O_3_ powders demonstrates that Ru was concentrated in certain zones of the Al_2_O_3_ powder support, as evidenced in Figure 3c,d. The SEM micrograph in Figure 3c shows that Ru is distributed in the interior the Al_2_O_3_ grains, likely in the pores, as well as irregularly segregated in the form of aggregates at certain regions, as indicated by arrows in the figure. This is also clearly seen in Figure 3d, showing EDX maps of the same powders demonstrating that, in given zones, Ru may form clusters within the alumina support. The presence of Ru at the surface of the Al_2_O_3_ support was also demonstrated by XPS analysis (see the Supporting Information). The size of Ru-particle aggregates has been estimated by performing a statistical analysis of TEM micrographs (using the ImageJ software) taken for different Ru-Al_2_O_3_ powder samples. This analysis renders a mean value of 120 ± 6 nm for the aggregates, the individual ruthenium nanoparticles having a much smaller size. This aggregate size is higher than that of nanoparticles prepared by using chemical reduction processes, as reported in the literature^43^ (see the Supporting Information). We should note that aggregation state of nanoparticles would not significantly affect their catalytic activity, which would be mainly determined by the density of surface-active catalysts sites, regardless of the aggregation state of the nanoparticles.

From the point of view of the potential catalytic activity and plasma behavior of the Ru-Al_2_O_3_/PZT pellets, it is noteworthy that Ru is not only located at the external surface of the pellets but also embedded within the Al_2_O_3_ particles and in its internal pores. This distribution is not equivalent to that commonly utilized in theoretical works to model the effect of the metallic phase in packed-bed systems, where metal particles are usually located at the external surface of the pellets.^44^

The BET surface area of the sintered PZT pellets was 0.7426 m^2^/g, while it was 3.9854 and 5.8014 m^2^/g for the Al_2_O_3_/PZT and Ru-Al_2_O_3_/PZT pellets, respectively. As expected, due to the alumina coating, the surface area of the covered PZT pellets (Al_2_O_3_/PZT and Ru-Al_2_O_3/_PZT configurations) increases, in agreement with the high surface area of the alumina powder. The difference between these two configurations is likely due to a different amount of coating material in each case.

### Electrical Behavior of the Packed-bed Reactor

**Figure 4:**
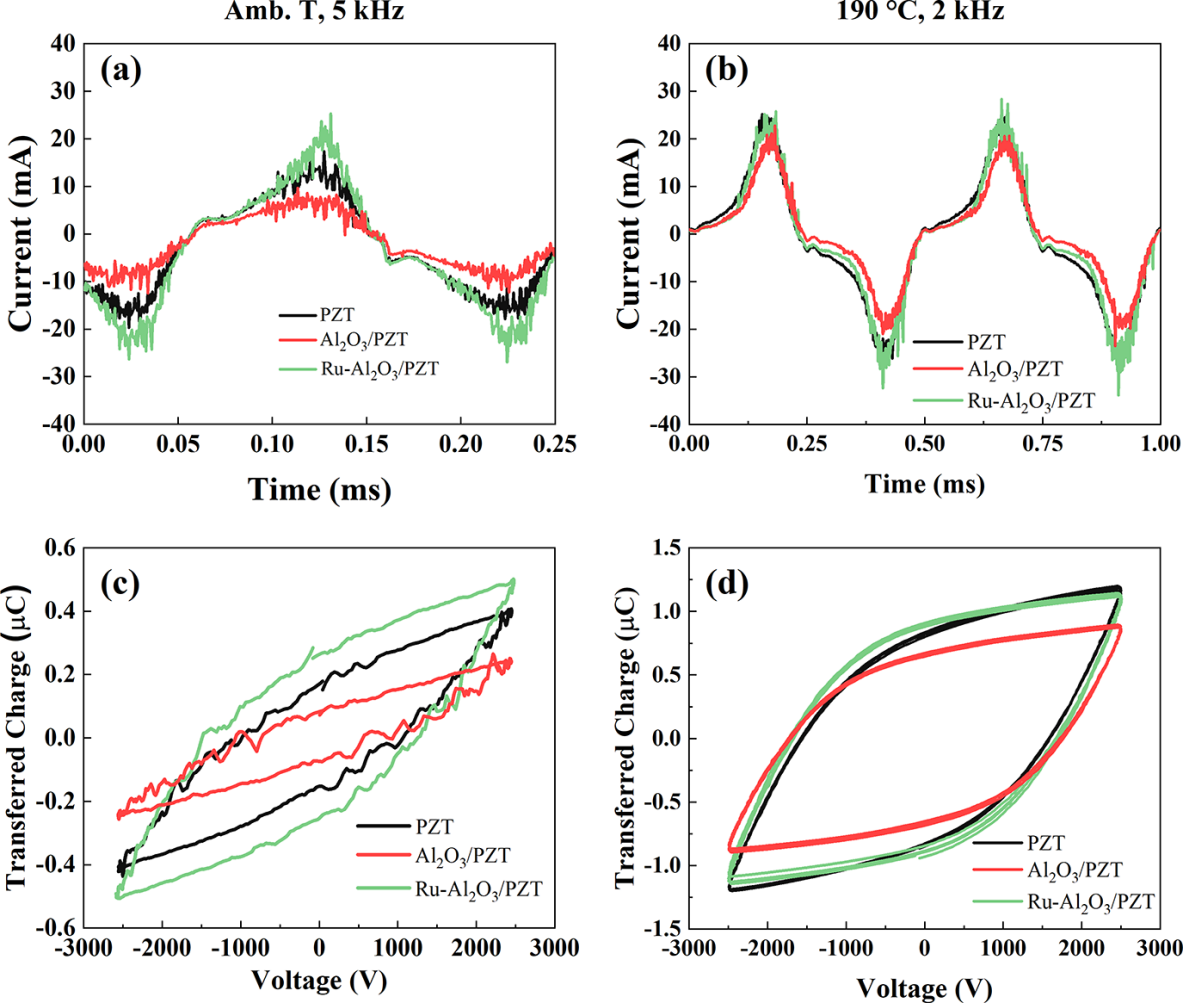
Electrical characterization of the reactor for the three barrier configurations (PZT, Al_2_O_3_/PZT, and Ru-Al_2_O_3_/PZT). *I*(*t*) curves during plasma ignition at (a) ambient temperature (2.5 kV, 5 kHz) and (b) 190 °C (2.5 kV, 2 kHz). (c) - (d) Lissajous plots determined from the curves in (a) and (b), respectively.

The characteristic *I*(*t*) curves measured at ambient temperature and at 190 °C were used to characterize the electrical behavior of the packed-bed reactor working in the PZT, Al_2_O_3_/PZT, and Ru-Al_2_O_3_/PZT configurations. Parts a and b of Figure 4 depict these curves recorded at 2.5 kV for experiments at ambient temperature (5 kHz) and 190 °C (2 kHz). Figure 4a shows that, although the three *I*(*t*) curves depict the typical microdischarge features of packed-bed reactors, the overall intensity varies at ambient temperature according to Ru-Al_2_O_3_/PZT > PZT > Al_2_O_3_/PZT. This is confirmed by the Lissajous plots depicted in Figure 4c, where it is apparent that the discharge power, taken as proportional to the area, is maximum for the reactor containing Ru-Al_2_O_3_/PZT pellets, followed by PZT and Al_2_O_3_/PZT pellets. The increase in plasma current found for the Ru-Al_2_O_3_/PZT configuration can be interpreted in the frame of recent modeling studies of plasma discharges taking place in metal particles decorating dielectric pellets: according to Kruszelnicki et al.,^44^ when metal particles are incorporated onto dielectric beads in packed-bed reactors operated at atmospheric pressure, there is an enhancement in plasma density in the proximity of the metal component. This effect was studied by the authors using a computational model and experimentally confirmed by iCCD imaging analysis. A similar effect could be expected for the Ru-Al_2_O_3_/PZT pellets, where Ru nanoparticles would act as plasma hot spots inducing a local change in the electric field and an increase in plasma electron density and energy.

It is noteworthy that the lower current and consumed power found for the Al_2_O_3_/PZT configuration agrees with the Comsol analysis discussed above, a feature that can be explained admitting that the Al_2_O_3_ coating, with a much lower dielectric constant than PZT, tends to *cool down* the plasma due to a decrease in the electric field intensity in the interpellet space. In line with these considerations, Lissajous figures reported in Figure 4c for the Al_2_O_3_/PZT configuration depict a smaller slope for both the cell (upper and lower lines in the figure) and the packing material capacitance (right and left side-lines), in agreement with the lower dielectric constant of Al_2_O_3_ as compared with PZT.^45^ Lissajous curves obtained at different voltages are shown in the Supporting Information, proving that plasma volume increases with the applied voltage.

The picture described in the previous paragraphs for the reactor operating at ambient temperature changed at 190 °C. In fact, unlike the relatively large differences in *I*(*t*) for the three configurations observed at ambient temperature, Figure 4b shows that the *I*(*t*) curves tend to overlap at 190 °C, with just slightly lower values for the Al_2_O_3_/PZT configuration. We attribute this behavior to the progressive nonlinear increase with temperature of the dielectric constant of PZT. In other words, the significantly higher value of the dielectric constant of PZT at 190 °C would be the predominant factor controlling the electrical response of the reactor. In agreement with this assessment, Lissajous plots shown in Figure 4d confirm that power consumption is similar for the three plasma reactor configurations when they are operated at 190 °C.

### Ammonia Synthesis at Ambient Temperature

**Figure 5:**
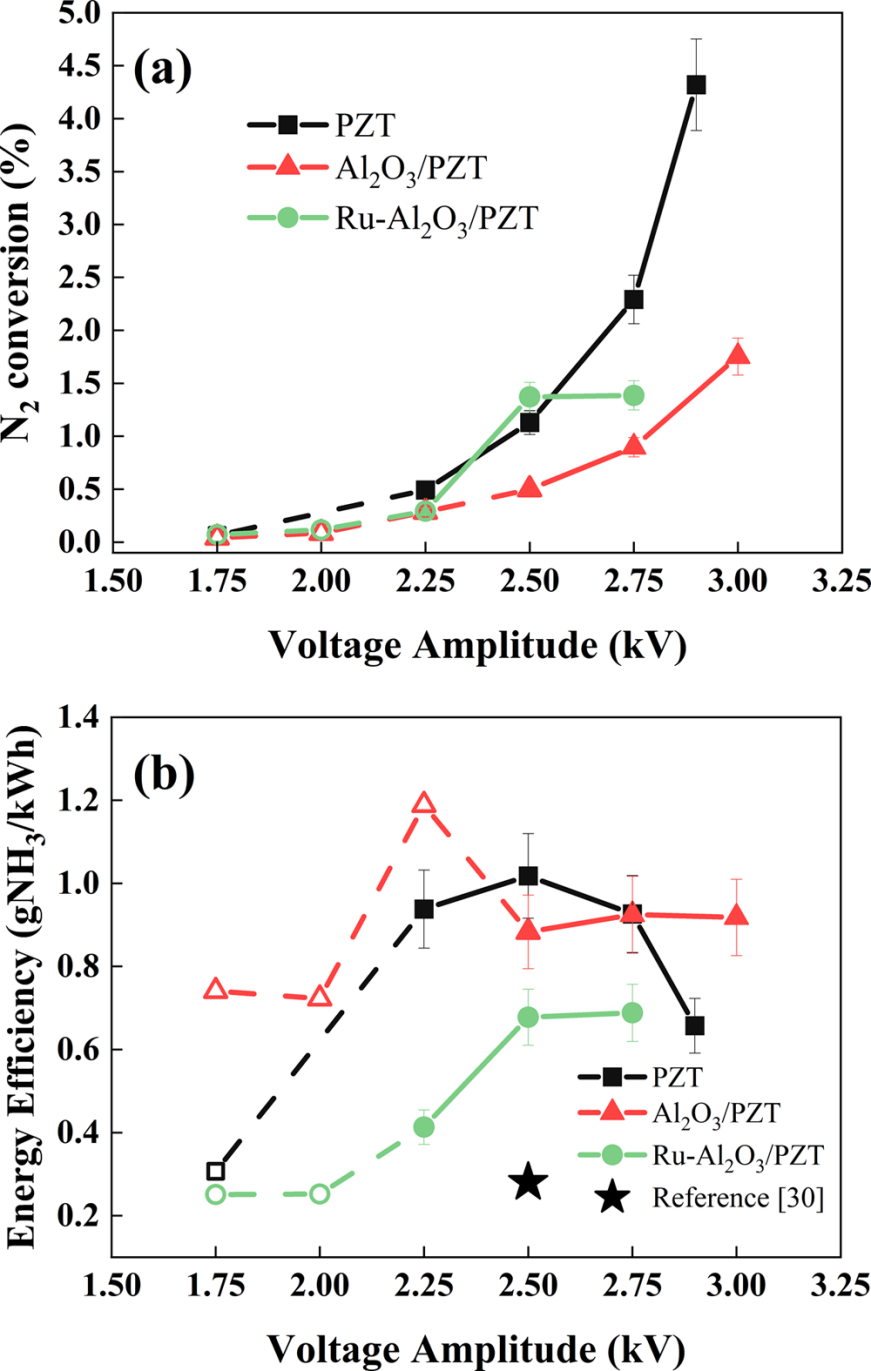
Evolution at ambient temperature of (a) the reaction yield and (b) the energy efficiency for the NH_3_ synthesis reaction as a function of the applied voltage. Data corresponding to low reaction yields are plotted with empty dots and dash lines. Experiments were carried out at a frequency of 5 kHz. The star dot corresponds to a 7% nitrogen conversion in experiments reported in ref (31).

The ammonia synthesis from N_2_+H_2_ plasmas has been first investigated at ambient temperature for the three barrier configurations of the packed-bed plasma reactor. Figure 5 shows the evolutions with the applied voltage of reaction yield (i.e., N_2_ conversion) and energy efficiency. According to Figure 5a, reaction yields smaller than 0.5% were obtained at low voltages for the three barriers. The uncertainty in accurately determining such little values of reaction yields and their correspondingly low consumed power values make the determination of the energy efficiency inaccurate. Accordingly, the points for these low-voltage experiments in Figure 5b are represented with empty dots joined by dashed lines to clearly distinguish these results from those that are meaningful for the analysis of the reactor performance. In this sense, the data in Figure 5a for voltages equal to and higher than 2.25 kV show that the reaction yield is higher for the reactor filled with PZT pellets. Interestingly, there is a similar high yield for the Ru-Al_2_O_3_/PZT configuration, except for the highest accessible voltage at which the conversion yield tends to decrease. The lowest yield values are obtained for the Al_2_O_3_/PZT reactor configuration, a result that can be related to the lower current intensity observed in the *I(t)* curves (cf. Figure 4a) and the lower electric field distribution between pellets deduced by COMSOL simulation (cf. Figure 2).

From a practical point of view, the energy efficiency of a plasma-catalysis process defines its actual capacity to compete with other established technologies.^46^ It is noteworthy in this regard that high conversion yields do not warrant high energy efficiencies and that an opposite evolution of these two magnitudes is a common behavior for different plasma catalysis processes.^12,47,48^ An example illustrating this tendency is included in Figure 5b. The star dot in the diagram corresponds to operating conditions taken from a previous study of our research group^31^ (i.e., a PZT barrier of 3 mm, flow rate of 11.5 sccm, frequency of 5 kHz, and voltage amplitude of 2.5 kV), where a N_2_ conversion yield of 7% was obtained. We realize here that this high conversion rate occurs at expenses of a relatively small energy efficiency in comparison with the conditions herein described, where a barrier of 5 mm includes a middle layer of pellets loaded with the Al_2_O_3_-supported metal catalyst (c.f., Figure 1).

The evolution of the energy efficiency with the applied voltage shown in Figure 5b reveals a different behavior depending on reactor configuration and suggests some differences in the reaction mechanisms. The Al_2_O_3_/PZT configuration depicts an approximately constant value of the energy efficiency for voltages higher than 2.25 kV, indicating that reaction mechanisms do not significantly change with the applied voltage. Meanwhile, the PZT barrier depicts an energy efficiency maximum at 2.5 kV, followed by a progressive small decrease at higher voltages. We tentatively attribute this decrease to the occurrence of inefficient processes such as the decomposition of ammonia and hydrogen exchange reactions.^21^ The tendency is similar for the Ru-Al_2_O_3_/PZT configuration and voltages higher than 2.25 kV, but always with lower efficiencies than for the PZT case. Since the reaction was carried out at ambient temperature, pure catalytic mechanisms induced by the Ru particles can be discarded. We propose that this systematic lower efficiency can be related to a higher probability of ammonia decomposition reactions induced by the impact with high-energy electrons generated in the microdischarges due to the presence of the metal phase (see in Figure 4a that there is a higher current amplitude for the Ru-Al_2_O_3_/PZT configuration).^44^ Meanwhile, for the Al_2_O_3_/PZT configuration, we assume that ammonia decomposition and/or hydrogen exchange reactions responsible to diminish the energy efficiency for the ammonia synthesis are less probable because of the claimed cooling down of the plasma for this configuration.

**Figure 6:**
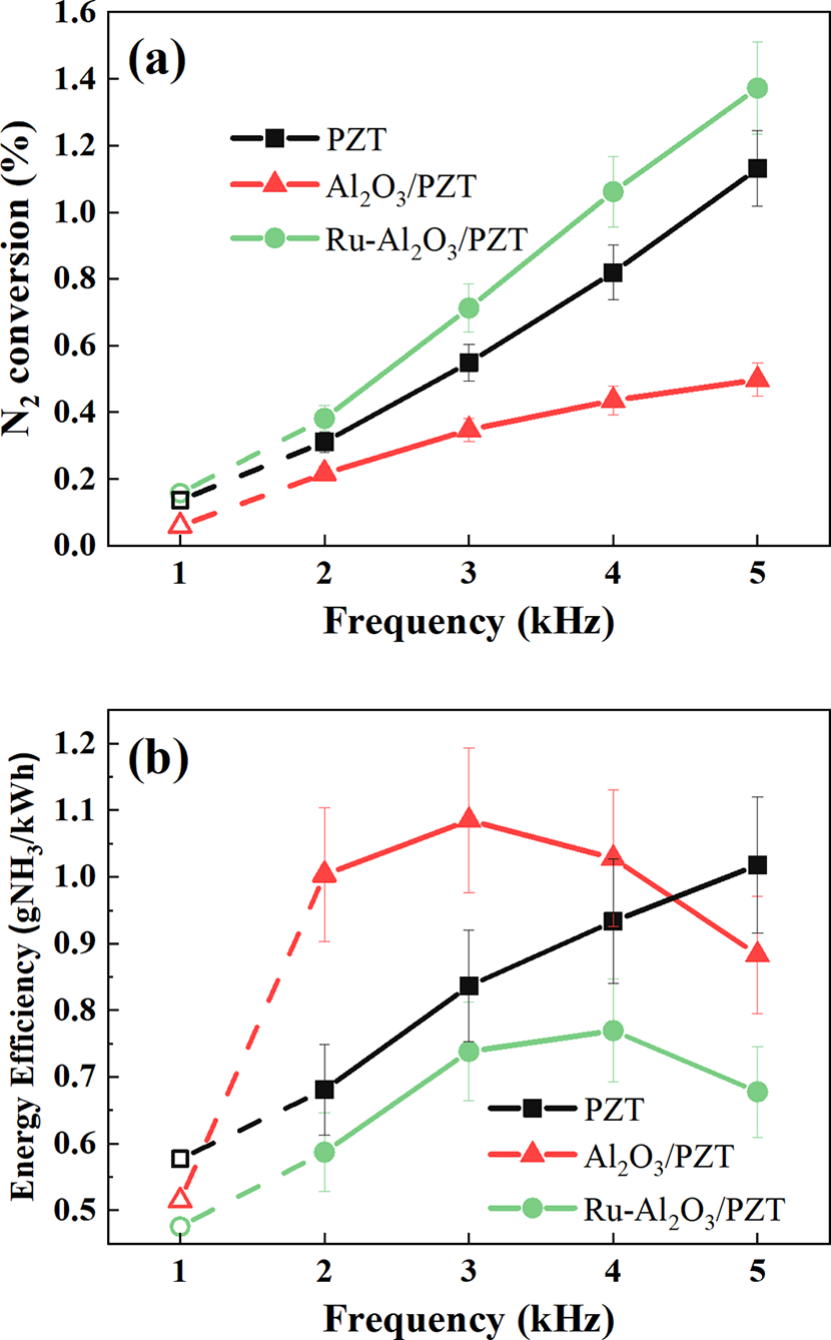
Evolution of (a) reaction yield and (b) energy efficiency for the ammonia synthesis reaction as a function of frequency. For the experiments at 1 kHz, results are plotted with empty dots and dash lines, indicating a possible inaccuracy in the determination of these values. Experiments were carried out at ambient temperature, a voltage amplitude of 2.5 kV, and a variable frequency between 1 and 5 kHz.

The previous considerations gain further credit examining the evolution of reaction yield and energy efficiency as a function of operating frequency (c.f., Figure 6). The frequency was varied systematically (1–5 kHz) at constant voltage (2.5 kV) as a way to modify the power consumed in the reactor (see the Supporting Information). Figure 6a shows that, in the three cases, the reaction yield progressively increases with frequency, although following different tendencies. The increment with the frequency is more noticeable in the case of Ru-Al_2_O_3_/PZT and PZT configurations, while in the Al_2_O_3_/PZT case, the slope of the curve is less pronounced, rendering values that follow the order Ru-Al_2_O_3_/PZT > PZT > Al_2_O_3_/PZT. Additionally, the evolution of the energy efficiency shown in Figure 6b reveals that this magnitude continuously increases for the PZT configuration, but it passes through a maximum (at around 2.5 kV) for the Ru-Al_2_O_3_/PZT one. Meanwhile, for the Al_2_O_3_/PZT configuration, the energy efficiency reaches a maximum at a frequency of 3 kHz. This behavior supports that, for the Ru-Al_2_O_3_/PZT configuration, inefficient reaction mechanisms (the reverse ammonia decomposition, reaction E1) or hydrogen exchange processes (reaction E2) are favored at increasing frequencies (i.e., consumed powers), something that is less evident for the PZT configuration, where the energy efficiency continuously increases with frequency. In this experiment, a maximum efficiency of 1g NH_3_/kWh was found at 5 kHz for the PZT configuration. According to the previous analysis, the maximum energy efficiency found for the Al_2_O_3_/PZT configuration at 3 kHz (1.3 g NH_3_/kWh) can be linked with a relatively lower probability of decomposition reactions under these conditions, or in other words, to that the power (or current) increase with frequency does not favor the occurrence of decomposition reactions for the Al_2_O_3_/PZT configuration.

### Ammonia Synthesis at Elevated Temperature

Within a plasma-catalysis perspective, a certain catalytic effect associated with the Ru particles might be expected at elevated temperatures.^37^ Therefore, similar experiments to those described in the previous section were carried out at a temperature of 190 °C (measured at the reactor wall). According to Rouwenhorst et al., this temperature is a kind of threshold for the catalytic promotion of the ammonia synthesis in plasma reactors incorporating a Ru-based catalyst.^37^ Owing to the specific conditions of our experiment, it is expected that the temperature inside the packed-bed zone may be higher that this nominal value at the reactor wall.^38^

**Figure 7:**
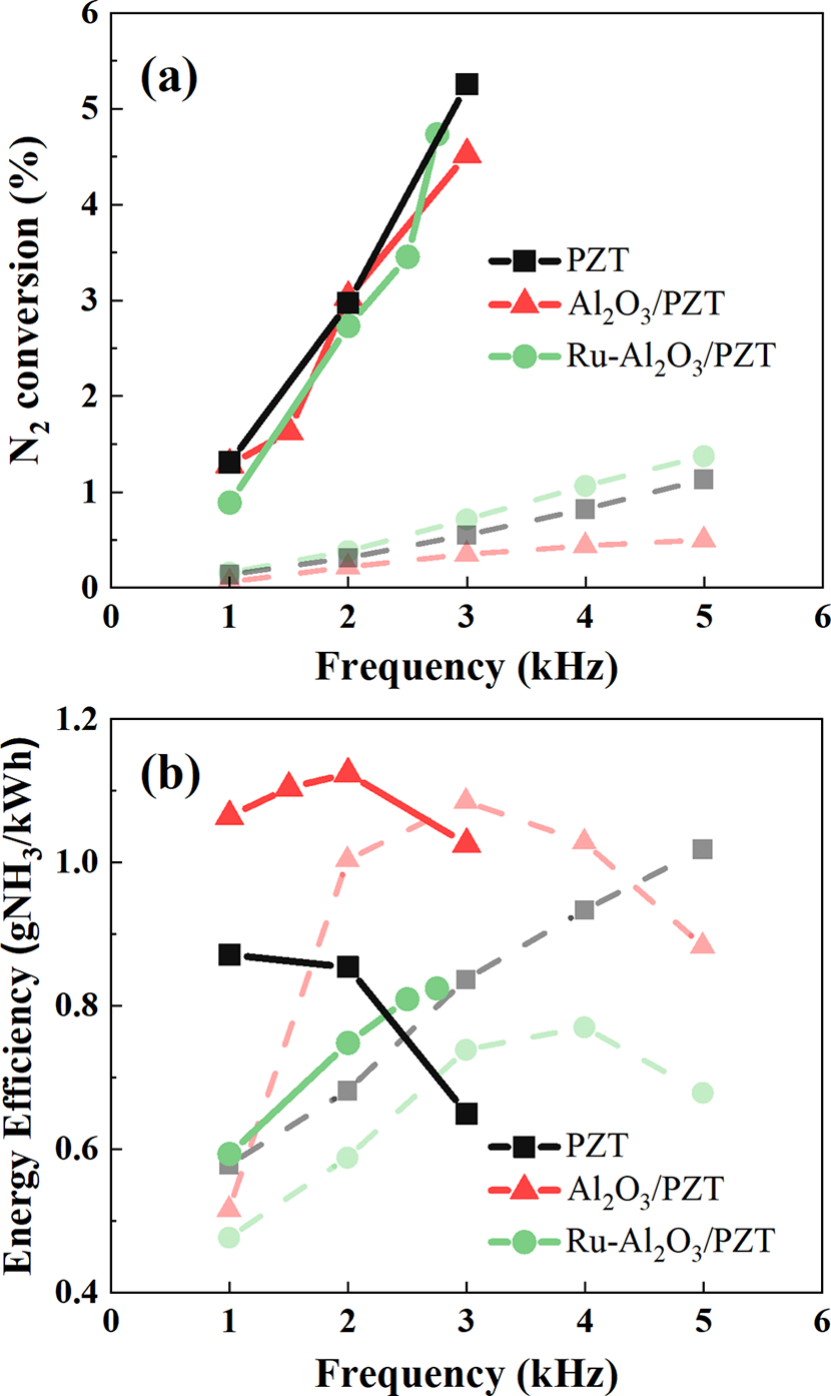
Evolution of (a) reaction yield and (b) energy efficiency for the ammonia synthesis reaction as a function of frequency. Experiments were carried out at 190 °C for a voltage amplitude of 2.5 kV and variable frequencies between 1 and 3 kHz. For comparison, data obtained under the same operating conditions at ambient temperature are included in the plot as dashed lines and faint colors.

Since an applied voltage of 2.5 kV provided the highest energy efficiency at ambient temperature (c.f., Figure 5), the 190 °C experiments were carried out at this voltage, varying the frequency between 1 and 3 kHz. The electrical characterization of the reactor under these operating conditions reported in Figure 4b,d, and in the Supporting Information shows that, at high temperature, current and power values increase with the frequency for all the configurations, although this increase is slightly less pronounced for the Al_2_O_3_/PZT configuration. Figure 7 shows the evolution with frequency of the reaction yield and energy efficiency at 190 °C (values obtained at ambient temperature, c.f. Figure 6b, are also plotted for comparative purposes). In Figure 7a, it can be observed that reaction yields significantly increase with respect to the yields obtained at ambient temperature, with values five times higher at 3 kHz and practically no differences between the three reactor configurations (in a similar way to the behavior found for the electrical response of the reactor). Remarkably, up to frequencies of 2 kHz, the energy efficiency increases (see Figure 7b), presenting significant differences depending on the configuration, following the order Ru-Al_2_O_3_/PZT < PZT < Al_2_O_3_/PZT. Then, for the PZT and Al_2_O_3_/PZT configurations, energy efficiency decreases at higher frequencies, while it remains still growing for the Ru-Al_2_O_3_/PZT configuration. We tentatively attribute the decrease in energy efficiency for the PZT configuration at 3 kHz to a progressive increase in the occurrence of inefficient processes (the mentioned hydrogen atom exchanges (2) and ammonia decomposition (1)). A similar small decrease is obtained at this frequency for the Al_2_O_3_/PZT configuration. Meanwhile, the different evolution found for the Ru-Al_2_O_3_/PZT configuration at frequencies higher than 2 kHz suggests changes in the reaction mechanisms, likely due to the appearance of new plasma-catalytic reaction pathways involving the Ru particles.^37^ In agreement with different authors, those reaction mechanisms could involve the dissociative adsorption of N_2_ molecules^23^ and the adsorption of vibrational excited nitrogen molecules that can interact with H or H_2_ species from the plasma (or adsorbed on the metal surface) to form adsorbed NH* species on the catalyst surface.^5,15^ However, the small differences found in reaction yield between the three barriers (c.f., Figure 7a) suggest that possible catalytic surface effects should be considered second-order or negligible with respect to other reaction pathways. This behavior differs from that of a typical catalytic process where the adsorption and surface dissociation of N_2_ and/or H_2_ molecules are required steps for the thermal catalytic synthesis of ammonia.^1,36,37,50,51^

For pure thermal catalytic processes, activation energies determined using Arrhenius plots are around 60–115 kJ/mol depending on the promoters used during the process.^51^ For the plasma-catalytic synthesis of ammonia, lower values found for different Ru-based catalysts are 20–40 kJ/mol.^52^ Claimed interpretation of this lower activation energy is that plasma contributes to excite the nitrogen molecule, favoring its dissociation on the catalyst site. Our results above suggest that this effect is negligible and that the electrical characteristic of the discharge is the most important factor affecting reaction efficiency. This view is also supported by previous results with PZT showing that the rate limiting step for the ammonia synthesis is the NH* formation in the plasma gas phase.^21^

As well as activation energy, turn over frequency (TOF) is a typical magnitude utilized to discuss catalytic efficiencies. It has been also used for plasma catalysis processes where values around 10^–3^–10^–2^ s^–1^ have been reported for the ammonia synthesis reaction.^52^ A rough calculation of TOF referred to the amount of Ru in the Ru-Al_2_O_3_/PZT pellets rendered values around 4 × 10^–4^ s^–1^, slightly lower than those obtained in these works. However, we would like to stress that calculation of TOF makes sense for cases where reactions take place at the surface of the catalysts. Our results above strongly suggest a negligible contribution of surface catalytic effects to the overall reaction process, disregarding such kind of calculations. A similar critical view has been maintained by other authors for plasma-catalysis processes.^22^

### OES Analysis: Intermediate Plasma Species at Ambient and Elevated Temperature

**Figure 8:**
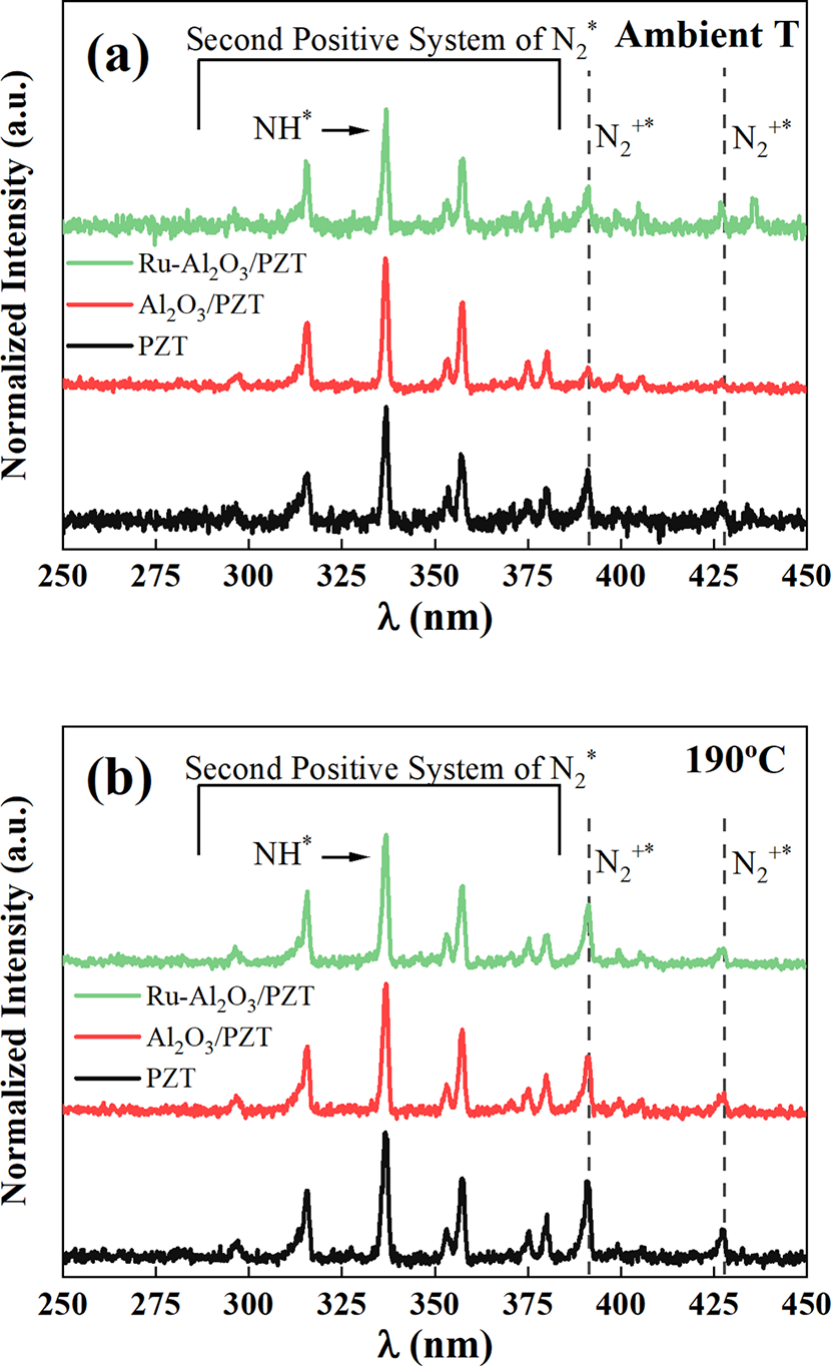
OES spectra recorded at (a) ambient temperature (2.5 kV, 5 kHz) and (b) 190 °C (2.5 kV, 2 kHz).

To further analyze the differences between the three configurations at ambient and high temperatures and gain insights on the reaction mechanisms, we have completed the previous study with optical emission measurements. Figure 8 shows the optical emission spectra acquired for the three configurations at (a) ambient temperature and (b) 190 °C. As observed, the following bands can be detected: NH* (transition [*A*^3^ Π → *X*^3^ ∑^–^] at 336 nm), the first negative system of N_2_^+^ (transition [*B*^2^ ∑_*u*_^+^ →*X*^2^ ∑_*g*_^+^], with main bands at 391.4 and 427.8 nm), and the second positive system of N_2_ (transition [*C*^3^ Π →*B*^3^ Π], with main bands at 337 and 357.9 nm).

Previous studies of our research group^29,31^ and other authors^18^ with N_2_ and H_2_ plasmas indicate that the presence of N_2_^+^ species in the gas phase is a required intermediate for the ammonia synthesis process, while NH* excited species can be associated with both synthesis and decomposition processes of ammonia. In particular, using the same reactor that in the present work and applying an isotope labeling methodology, we demonstrated that a pure nitrogen plasma does not render ammonia molecules, even if the pellet surface is previously saturated with hydrogen atoms. To obtain ammonia molecules it is necessary to add hydrogen to the gas phase.^21^ Thus, we propose that the formation of NH* species in the plasma is a rate-limiting step for the ammonia synthesis. Based on these premises on the role of detected plasma species, we propose the following plasma mechanistic scheme for the synthesis of ammonia**:**
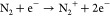
3
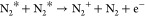
4
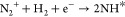
5
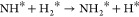
6
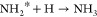
7

Note that hydrogenation (reactions 5–7) might take place in the plasma gas phase, on the surface of the packed material (i.e., through an L–H mechanisms) or also through the interaction of NH* species from the plasma with H atoms adsorbed on the surface of metal particles or exposed zones of PZT (E–R mechanisms). L–H and E–R mechanisms involving Ru nanoparticles entail a surface catalytic process, which we consider negligible in our case. Although no emission lines corresponding to atomic nitrogen are detected by OES, the formation of NH* in the plasma phase could also start from processes such as reaction 8 or 9:
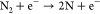
8
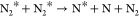
9

Looking at the spectra in Figure 8, at ambient and elevated temperatures, there are slightly differences in the ratio between bands and therefore in the relative concentration of species in the plasma phase. At first glance, it is observed that band associated with the N_2_^+^ at 190 °C presents a higher intensity that those obtained at room temperature and that, for both cases (room and elevated temperature), the N_2_^+^ band intensity measured for the Al_2_O_3_/PZT configuration is smaller than for the other two configurations (PZT and Ru-Al_2_O_3_/PZT). To analyze this fact, we evaluate the normalized N_2_^+^ relative emission intensity, i.e., the N_2_^+^/N_2_* ratio, by supposing that the ration between band height is proportional to the ratio between the concentration of the excited emitting species. At ambient temperature (5 kHz, 2.5 kV), the intensity ratios were 0.90, 0.49, and 0.82 for the PZT, Al_2_O_3_/PZT, and Ru-Al_2_O_3_/PZT configurations, respectively, while values of 0.98, 0.75, and 0.87 were found at 190 °C (2 kHz, 2.5 kV). Most remarkable is that at ambient temperature the N_2_^+^ relative emission intensity was smaller for the Al_2_O_3_/PZT configuration. Since the formation of N_2_^+^ species requires electrons with energies equal or higher than 15.6 eV,^53^ the lower population of this excited specie agrees with the claimed cooling down effect of plasma due to the alumina coating (c.f., Figures 2 and 4a,c) in the Al_2_O_3_/PZT configuration. A consequence of this cooling effect would also be the decrease in reaction yield obtained at 5 kHz at ambient temperature and at 2 kHz at 190 °C for the Al_2_O_3_/PZT (0.5%) with respect to the other configurations (around 1.3%) (c.f., Figure 6a). For these two operating conditions, the efficiency of the Al_2_O_3_ configuration tends to decrease, although still maintaining a higher energy efficiency than for the other the operating conditions, especially at high temperatures. The reduction in the mean electron energy due to the lower electric field intensity obtained when incorporating the alumina coating might also contribute to decrease the impact of back-reactions (i.e., ammonia decomposition) and therefore to increase the generally higher energy efficiency found for the Al_2_O_3_/PZT configuration.

On the other hand, the similar N_2_^+^/N_2_* intensity ratios obtained at high temperatures for the three barrier configurations suggest that, for the essayed operating conditions, catalytic reaction pathways do not significantly contribute to the reaction, even in the presence of ruthenium particles. The similar nitrogen conversion rates determined for the three barriers (c.f., Figure 7a) also support this assessment. Only the growing tendency in energy efficiency reported in Figure 7b for the Ru-Al_2_O_3_/PZT configuration allows us to think about a certain catalytic effect associated with the Ru particles, although its magnitude should be very small or negligible. Indeed, the similar conversions found for the three configurations and the higher efficiencies (see Figure 7) found for the PZT and Al_2_O_3_/PZT configurations support this view.

According to these inferences, we propose that rather than to a catalytic effect of the metal particles (not discarded, but comparatively negligible), the rather high reaction yield and energy efficient values obtained with the three configurations at 190 °C (generally higher than recently reported values from the literature, see, for example, review publications in refs (6 and 54)), should be primarily attributed to the enhancement of plasma intensity induced by the PZT ferroelectric barrier and the variation of its electrical behavior when increasing the temperature of the barrier.^38^

## Conclusions

In this study, three different barrier configurations (PZT, Al_2_O_3_/PZT, and Ru-Al_2_O_3_/PZT) have been systematically essayed to study the effect of the incorporation of a ruthenium metal catalyst in a ferroelectric packed-bed reactor for the synthesis of ammonia. Ferroelectric packed-bed reactors are characterized by high plasma currents due to the high dielectric constant of the ferroelectric PZT used as moderator materials. The operation of plasma reactors moderated with ferroelectric materials, both at ambient and high temperatures, has allowed us to conclude that metal catalyst are not particularly beneficial for the ammonia synthesis under the experimental conditions herein analyzed, even at high temperatures at which a catalytic activity has been proposed in the literature to contribute to the formation of ammonia. We relate that the apparent lack of a beneficial catalytic activity to that NH_3_ decomposition can be also promoted by interactions with the high-energy electrons formed in the high-intense plasma microdischarges induced by the metal particles. We should remark that this enhancement of detrimental plasma reactions does not discard that the ammonia synthesis reaction may be favored by pure catalytically driven processes occurring at the surface of the metal catalyst. However, the overall consequence is that the final production of ammonia is similar for the three configurations and that the energy efficiency is always smaller for Ru-Al_2_O_3_/PZT than for the Al_2_O_3_/PZT configuration and, for certain values of frequency, also for the PZT configuration.

It has been also demonstrated that a pristine alumina coating covering the PZT pellets tends to cool down the plasma due to the decrease in the electric field intensity in the interpellet space. Additionally, we propose that the alumina coating can decrease the occurrence of inefficient reactions taking place at the PZT surface at elevated temperatures. As a general conclusion, unlike the claims of different studies reported in the literature using dielectrics as moderators,^22,23,25,35−37^ the incorporation of metal catalysts—even activated at high temperatures—in ferroelectric packed-bed reactors does not seem to be the best strategy to improve the plasma-catalytic performance of the ammonia synthesis reaction.

## Supporting Information Available

S1 - Detailed description of the experimental apparatus, S2 - Comsol Multiphysics simulations of PZT pellets, S3 - XPS analysis of the Al_2_O_3_/PZT sample, S4 - XPS analysis and TEM micrographs of Al_2_O_3_ and Ru-Al_2_O_3_ powder samples, S5 - Lissajous plots at different applied voltages, and S6 - Evolution of the consumed power with the frequency at ambient temperature and S7 - at 190 °C (PDF)

## References

[ref1] Smith C.; Hill A. K.; Torrente-Murciano L. Current and Future Role of Haber-Bosch Ammonia in a Carbon-Free Energy Landscape. Energy Environ. Sci. 2020, 13 (2), 331–344. 10.1039/C9EE02873K.

[ref2] Ertl G. Reactions at Surfaces: From Atoms to Complexity (Nobel Lecture). Angew. Chem., Int. Ed. 2008, 47 (19), 3524–3535. 10.1002/anie.200800480. 18357601

[ref3] Liu X.; Elgowainy A.; Wang M. Life Cycle Energy Use and Greenhouse Gas Emissions of Ammonia Production from Renewable Resources and Industrial By-Products. Green Chem. 2020, 22 (17), 5751–5761. 10.1039/D0GC02301A.

[ref4] Rouwenhorst K. H. R.; Engelmann Y.; van ‘t Veer K.; Postma R. S.; Bogaerts A.; Lefferts L. Plasma-Driven Catalysis: Green Ammonia Synthesis with Intermittent Electricity. Green Chem. 2020, 22 (19), 6258–6287. 10.1039/D0GC02058C.

[ref5] Wang Y.; Yang W.; Xu S.; Zhao S.; Chen G.; Weidenkaff A.; Hardacre C.; Fan X.; Huang J.; Tu X. Shielding Protection by Mesoporous Catalysts for Improving Plasma-Catalytic Ambient Ammonia Synthesis. J. Am. Chem. Soc. 2022, 144, 12020 10.1021/jacs.2c01950. 35731953 PMC9284550

[ref6] Carreon M. L. Plasma Catalytic Ammonia Synthesis: State of the Art and Future Directions. J. Phys. D: Appl. Phys. 2019, 52 (48), 483001 10.1088/1361-6463/ab3b2c.

[ref7] Wu A.; Yang J.; Xu B.; Wu X. Y.; Wang Y.; Lv X.; Ma Y.; Xu A.; Zheng J.; Tan Q.; Peng Y.; Qi Z.; Qi H.; Li J.; Wang Y.; Harding J.; Tu X.; Wang A.; Yan J.; Li X. Direct Ammonia Synthesis from the Air via Gliding Arc Plasma Integrated with Single Atom Electrocatalysis. Appl. Catal., B 2021, 299, 120667 10.1016/j.apcatb.2021.120667.

[ref8] Gorbanev Y.; Vervloessem E.; Nikiforov A.; Bogaerts A. Nitrogen Fixation with Water Vapor by Nonequilibrium Plasma: Toward Sustainable Ammonia Production. ACS Sustain Chem. Eng. 2020, 8 (7), 2996–3004. 10.1021/acssuschemeng.9b07849.

[ref9] Antunes R.; Marot L.; Romero-Muniz C.; Steiner R.; Meyer E. The Role of Tungsten Chemical State and Boron on Ammonia Formation Using N_2_–H_2_ Radiofrequency Discharges. Nucl. Fusion 2021, 61 (12), 126046 10.1088/1741-4326/ac33c6.

[ref10] Bogaerts A.; Tu X.; Whitehead J. C.; Centi G.; Lefferts L.; Guaitella O.; Azzolina-Jury F.; Kim H. H.; Murphy A. B.; Schneider W. F.; Nozaki T.; Hicks J. C.; Rousseau A.; Thevenet F.; Khacef A.; Carreon M. The 2020 Plasma Catalysis Roadmap. J. Phys. D: Appl. Phys. 2020, 53 (44), 443001 10.1088/1361-6463/ab9048.

[ref11] Yan C.; Waitt C.; Akintola I.; Lee G.; Easa J.; Clarke R.; Geng F.; Poirier D.; Otor H. O.; Rivera-Castro G.; Go D. B.; O’Brien C. P.; Hicks J. C.; Schneider W. F.; Ma H. Recent Advances in Plasma Catalysis. J. Phys. Chem. C 2022, 126 (23), 9611–9614. 10.1021/acs.jpcc.2c03062.

[ref12] Liu J.; Zhu X.; Zhou C.; Du J.; Gan Y.; Chen G.; Tu X. Plasma-Catalytic Ammonia Synthesis over BaTiO_3_ Supported Metal Catalysts: Process Optimization Using Response Surface Methodology. Vacuum 2022, 203, 111205 10.1016/j.vacuum.2022.111205.

[ref13] Zhu X.; Liu J.; Hu X.; Zhou Z.; Li X.; Wang W.; Wu R.; Tu X. Plasma-Catalytic Synthesis of Ammonia over Ru-Based Catalysts: Insights into the Support Effect. Journal of the Energy Institute 2022, 102, 240–246. 10.1016/j.joei.2022.02.014.

[ref14] Gorky F.; Lucero J. M.; Crawford J. M.; Blake B. A.; Guthrie S. R.; Carreon M. A.; Carreon M. L. Insights on Cold Plasma Ammonia Synthesis and Decomposition Using Alkaline Earth Metal-Based Perovskites. Catal. Sci. Technol. 2021, 11 (15), 5109–5118. 10.1039/D1CY00729G.

[ref15] Liu T.-W.; Gorky F.; Carreon M. L.; Gómez-Gualdrón D. A. Energetics of Reaction Pathways Enabled by N and H Radicals during Catalytic, Plasma-Assisted NH_3_ Synthesis. ACS Sustain Chem. Eng. 2022, 10 (6), 2034–2051. 10.1021/acssuschemeng.1c05660.

[ref16] Gorky F.; Best A.; Jasinski J.; Allen B. J.; Alba-Rubio A. C.; Carreon M. L. Plasma Catalytic Ammonia Synthesis on Ni Nanoparticles: The Size Effect. J. Catal. 2021, 393, 369–380. 10.1016/j.jcat.2020.11.030.

[ref17] Akay G.; Zhang K. Process Intensification in Ammonia Synthesis Using Novel Coassembled Supported Microporous Catalysts Promoted by Nonthermal Plasma. Ind. Eng. Chem. Res. 2017, 56 (2), 457–468. 10.1021/acs.iecr.6b02053.

[ref18] Gorky F.; Lucero J. M.; Crawford J. M.; Blake B.; Carreon M. A.; Carreon M. L. Plasma-Induced Catalytic Conversion of Nitrogen and Hydrogen to Ammonia over Zeolitic Imidazolate Frameworks ZIF-8 and ZIF-67. ACS Appl. Mater. Interfaces 2021, 13 (18), 21338–21348. 10.1021/acsami.1c03115. 33908750

[ref19] Gorky F.; Guthrie S. R.; Smoljan C. S.; Crawford J. M.; Carreon M. A.; Carreon M. L. Plasma Ammonia Synthesis over Mesoporous Silica SBA-15. J. Phys. D Appl. Phys. 2021, 54 (26), 264003 10.1088/1361-6463/abefbc.

[ref20] Rouwenhorst K. H. R.; Mani S.; Lefferts L. Improving the Energy Yield of Plasma-Based Ammonia Synthesis with In Situ Adsorption. ACS Sustain Chem. Eng. 2022, 10 (6), 1994–2000. 10.1021/acssuschemeng.1c08467.

[ref21] Navascués P.; Obrero-Pérez J. M.; Cotrino J.; González-Elipe A. R.; Gómez-Ramírez A. Unraveling Discharge and Surface Mechanisms in Plasma-Assisted Ammonia Reactions. ACS Sustain Chem. Eng. 2020, 8 (39), 14855–14866. 10.1021/acssuschemeng.0c04461.

[ref22] Engelmann Y.; Van’T Veer K.; Gorbanev Y.; Neyts E. C.; Schneider W. F.; Bogaerts A. Plasma Catalysis for Ammonia Synthesis: A Microkinetic Modeling Study on the Contributions of Eley-Rideal Reactions. ACS Sustain Chem. Eng. 2021, 9 (39), 13151–13163. 10.1021/acssuschemeng.1c02713.

[ref23] Mehta P.; Barboun P.; Herrera F. A.; Kim J.; Rumbach P.; Go D. B.; Hicks J. C.; Schneider W. F. Overcoming Ammonia Synthesis Scaling Relations with Plasma-Enabled Catalysis. Nature Catalysis 2018 1:4 2018, 1 (4), 269–275. 10.1038/s41929-018-0045-1.

[ref24] Mehta P.; Barboun P. M.; Engelmann Y.; Go D. B.; Bogaerts A.; Schneider W. F.; Hicks J. C. Plasma-Catalytic Ammonia Synthesis beyond the Equilibrium Limit. ACS Catal. 2020, 10 (12), 6726–6734. 10.1021/acscatal.0c00684.

[ref25] Gorbanev Y.; Engelmann Y.; Van’T Veer K.; Vlasov E.; Ndayirinde C.; Yi Y.; Bals S.; Bogaerts A. Al_2_O_3_-Supported Transition Metals for Plasma-Catalytic NH_3_ Synthesis in a DBD Plasma: Metal Activity and Insights into Mechanisms. Catalysts 2021, 11 (10), 1230 10.3390/catal11101230.

[ref26] Carreon M. L. Plasma Catalysis: A Brief Tutorial. Plasma Research Express 2019, 1 (4), 043001 10.1088/2516-1067/ab5a30.

[ref27] Wang X.; Xu S.; Yang W.; Fan X.; Pan Q.; Chen H. Development of Ni-Co Supported on SBA-15 Catalysts for Non-Thermal Plasma Assisted Co-Conversion of CO_2_ and CH_4_: Results and Lessons Learnt. Carbon Capture Science & Technology 2022, 5, 100067 10.1016/j.ccst.2022.100067.

[ref28] Bogaerts A.; Neyts E. C.; Guaitella O.; Murphy A. B. Foundations of Plasma Catalysis for Environmental Applications. Plasma Sources Sci. Technol. 2022, 31 (5), 053002 10.1088/1361-6595/ac5f8e.

[ref29] Gómez-Ramírez A.; Cotrino J.; Lambert R. M.; González-Elipe A. R. Efficient Synthesis of Ammonia from N_2_ and H_2_ Alone in a Ferroelectric Packed-Bed DBD Reactor. Plasma Sources Sci. Technol. 2015, 24 (6), 065011 10.1088/0963-0252/24/6/065011.

[ref31] Gómez-Ramírez A.; Montoro-Damas A. M.; Cotrino J.; Lambert R. M.; González-Elipe A. R. About the Enhancement of Chemical Yield during the Atmospheric Plasma Synthesis of Ammonia in a Ferroelectric Packed Bed Reactor. Plasma Processes Polym. 2017, 14 (6), 1600081 10.1002/ppap.201600081.

[ref32] Montoro-Damas A. M.; Brey J. J.; Rodríguez M. A.; González-Elipe A. R.; Cotrino J. Plasma Reforming of Methane in a Tunable Ferroelectric Packed-Bed Dielectric Barrier Discharge Reactor. J. Power Sources 2015, 296, 268–275. 10.1016/j.jpowsour.2015.07.038.

[ref33] Gómez-Ramírez A.; Montoro-Damas A.; Rodríguez M. A.; González-Elipe A.; Cotrino J. Improving the Pollutant Removal Efficiency of Packed-Bed Plasma Reactors Incorporating Ferroelectric Components. Chem. Eng. J. 2017, 314, 311–319. 10.1016/j.cej.2016.11.065.

[ref34] Navascués P.; Cotrino J.; González-Elipe A. R.; Gómez-Ramírez A. Plasma Assisted CO_2_ Dissociation in Pure and Gas Mixture Streams with a Ferroelectric Packed-Bed Reactor in Ambient Conditions. Chem. Eng. J. 2022, 430, 133066 10.1016/j.cej.2021.133066.

[ref35] Hu X.; Zhu X.; Wu X.; Cai Y.; Tu X. Plasma-Enhanced NH_3_ Synthesis over Activated Carbon-Based Catalysts: Effect of Active Metal Phase. Plasma Processes Polym. 2020, 17 (12), 2000072 10.1002/ppap.202000072.

[ref36] Rouwenhorst K. H. R.; Lefferts L. On the Mechanism for the Plasma-Activated N_2_ dissociation on Ru Surfaces. J. Phys. D: Appl. Phys. 2021, 54 (39), 393002 10.1088/1361-6463/ac1226.

[ref37] Rouwenhorst K. H. R.; Burbach H. G. B.; Vogel D. W.; Núñez Paulí J.; Geerdink B.; Lefferts L. Plasma-Catalytic Ammonia Synthesis beyond Thermal Equilibrium on Ru-Based Catalysts in Non-Thermal Plasma. Catal. Sci. Technol. 2021, 11 (8), 2834–2843. 10.1039/D0CY02189J.

[ref38] Gómez-Ramírez A.; Álvarez R.; Navascués P.; García-García F. J.; Palmero A.; Cotrino J.; González-Elipe A. R. Electrical and Reaction Performances of Packed-Bed Plasma Reactors Moderated with Ferroelectric or Dielectric Materials. Plasma Processes Polym. 2021, 18 (3), 2000193 10.1002/ppap.202000193.

[ref39] Patil B. S. Plasma (Catalyst) - Assisted Nitrogen Fixation : Reactor Development for Nitric Oxide and Ammonia Production. PhD Thesis, Technische Universiteit Eindhoven, 2017.

[ref40] COMSOL Multiphysics v.5.5; COMSOL AB: Stockholm, Sweden, 2019.

[ref41] van Laer K.; Bogaerts A. How Bead Size and Dielectric Constant Affect the Plasma Behaviour in a Packed Bed Plasma Reactor: A Modelling Study. Plasma Sources Sci. Technol. 2017, 26 (8), 085007 10.1088/1361-6595/aa7c59.

[ref42] van Laer K.; Bogaerts A. Fluid Modelling of a Packed Bed Dielectric Barrier Discharge Plasma Reactor. Plasma Sources Sci. Technol. 2016, 25 (1), 015002 10.1088/0963-0252/25/1/015002.

[ref43] Krajczewski J.; Ambroziak R.; Kudelski A. Formation and Selected Catalytic Properties of Ruthenium, Rhodium, Osmium and Iridium Nanoparticles. RSC Adv. 2022, 12 (4), 2123–2144. 10.1039/D1RA07470A.

[ref44] Kruszelnicki J.; Engeling K. W.; Foster J. E.; Kushner M. J. Interactions between Atmospheric Pressure Plasmas and Metallic Catalyst Particles in Packed Bed Reactors. J. Phys. D Appl. Phys. 2021, 54 (10), 104001 10.1088/1361-6463/abcc92.

[ref45] Peeters F. J. J.; van de Sanden M. C. M. The Influence of Partial Surface Discharging on the Electrical Characterization of DBDs. Plasma Sources Sci. Technol. 2015, 24 (1), 015016 10.1088/0963-0252/24/1/015016.

[ref46] Rouwenhorst K. H. R.; Lefferts L. Feasibility Study of Plasma-Catalytic Ammonia Synthesis for Energy Storage Applications. Catalysts 2020, Vol. 10, Page 999 2020, 10 (9), 999 10.3390/catal10090999.

[ref47] Uytdenhouwen Y.; Meynen V.; Cool P.; Bogaerts A. The Potential Use of Core-Shell Structured Spheres in a Packed-Bed DBD Plasma Reactor for CO_2_ Conversion. Catalysts 2020, 10 (5), 530 10.3390/catal10050530.

[ref48] Whitehead J. C. Plasma Catalysis: Introduction and History. In Plasma Catalysis. Fundamentals and Applications; Tu X., Whitehead J. C., Nozaki T., Eds.; Springer, Cham, 2019; Vol. 1, pp 1–19. 10.1007/978-3-030-05189-1_1

[ref50] Bell T. E.; Torrente-Murciano L. H_2_ Production via Ammonia Decomposition Using Non-Noble Metal Catalysts: A Review. Top Catal 2016, 59 (15–16), 1438–1457. 10.1007/s11244-016-0653-4.

[ref51] Rouwenhorst K. H. R.; Kim H.-H.; Lefferts L. Vibrationally Excited Activation of N_2_ in Plasma-Enhanced Catalytic Ammonia Synthesis: A Kinetic Analysis. *ACS Sustain*. Chem. Eng. 2019, 7 (20), 17515–17522. 10.1021/acssuschemeng.9b04997.

[ref52] Rouwenhorst K. H. R. Nitrogen Fixation with Renewable Electricity: Plasma Catalysis as an Alternative for Small-Scale Ammonia Synthesis?; University of Twente: Enschede, The Netherlands, 2022. 10.3990/1.9789036554190.

[ref53] Trickl T.; Cromwell E. F.; Lee Y. T.; Kung A. H. State-selective Ionization of Nitrogen in the X 2∑+gv+=0 and V+=1 States by Two-color (1+ 1) Photon Excitation near Threshold. J. Chem. Phys. 1989, 91 (10), 6006 10.1063/1.457417.

[ref54] Zhou D.; Zhou R.; Zhou R.; Liu B.; Zhang T.; Xian Y.; Cullen P. J.; Lu X.; Ostrikov K. Sustainable Ammonia Production by Non-Thermal Plasmas: Status, Mechanisms, and Opportunities. Chem. Eng. J. 2021, 421, 129544 10.1016/j.cej.2021.129544.

