# Supplementary material for: Incorporation of a Metal Catalyst for the Ammonia Synthesis in a Ferroelectric Packed-Bed Plasma Reactor: Does It Really Matter?
Source: ACS Sustain Chem Eng. 2023 Feb 20;11(9):3621–32. doi: 10.1021/acssuschemeng.2c05877 (PMC9993574; doi:10.1021/acssuschemeng.2c05877)
Supplement: Supplementary file 1 — sc2c05877_si_001.pdf [file sc2c05877_si_001.pdf]

The incorporation of a metal catalyst for the ammonia synthesis in a ferroelectric packed-bed plasma reactor: does it really matter?

Paula Navascués,*[a] Juan Garrido-García,[a] José Cotrino,[a,b] Agustín R. González-Elipe [a] and Ana Gómez-Ramírez*[a,b]

[a] Laboratory of Nanotechnology on Surfaces and Plasma. Instituto de Ciencia de Materiales de Sevilla (CSIC-Universidad de Sevilla), Avda. Américo Vespucio 49, E-41092 Seville, Spain.
[b] Departamento de Física Atómica, Molecular y Nuclear, Universidad de Sevilla, Avda. Reina Mercedes, E-41012 Seville, Spain
* Corresponding authors
anamgr@us.es
paula.navascues@icmse.csic.es

This SI contains 9 pages from S1 to S7, 7 figures, and 0 tables.

Supporting Information S1. Detailed description of the experimental apparatus.

Figure S1 illustrates a sketch of the packed-bed reactor and its connection with the power supply and the analytical system. Analytical techniques include Optical Emission Spectroscopy (OES), Electrical Characterization, and Quadrupole Mass Spectrometry (QMS). As indicated in the main text, the packed-bed plasma reactor, made entirely of stainless steel, consists of two parallel-plate electrodes separated 5 mm by packed ferroelectric pellets.

The gas inlet mixture was introduced into the reactor through a hole located in the center of the bottom electrode, while the outlet gas mixture left the reactor chamber through two exhausts located in the upper part. The gas flow rate was monitored with mass flow controllers (MFC, Bronkhorst, Nederland).

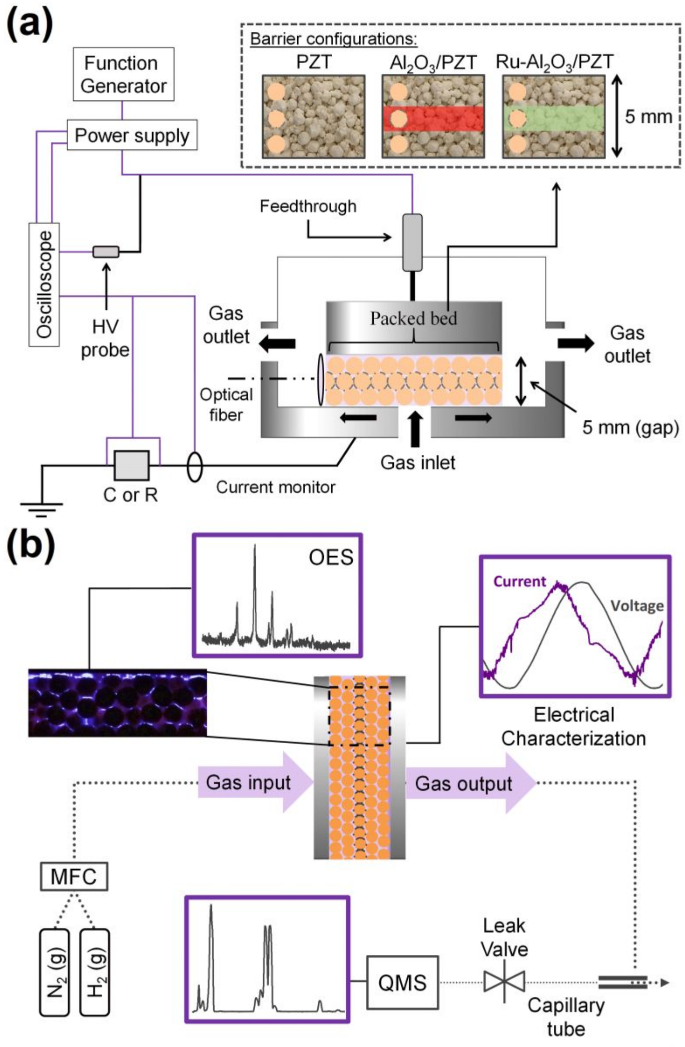

Figure S1. (a) Sketch of the reaction system (packed-bed reactor & electrical excitation system). (b) Experimental setup indicating the connections between the gas supply, the reactor, and the analytical system used to follow up the plasma-driven ammonia reactions.

Supporting Information S2. Comsol Multiphysics simulations of PZT pellets.

To simulate the pellets topography, we have built random curves in 2D by using spatial oscillations. These oscillations are quite similar to temporal oscillations used in electromagnetism: *cos(2πft)*, where *f* is the frequency (s^{-1}) and *t* the time (s). Spatial oscillations can be given by *cos(2πνx)*, where *ν* is the spatial frequency (m^{-1}) and *x* is the position (m). Taking into account that the wave number can be written as $k = 2\pi\nu$, these spatial waves are easily generalizable to a space with more than one spatial frequency (two in our case). So, we can write the equations as follows:

$$\cos(\vec{k} * \vec{r}\,) = \cos(2\pi(\nu_x x + \nu_y y); \vec{k} = (k_x, k_y), \vec{r} = (x, y) \qquad (1)$$

By using elemental waves we can describe a random surface *f*(*x,y*) as a superposition of different waves with a form defined by:

$$\cos\left(\vec{k} * \vec{r} + \phi\right) \qquad (2)$$

where *Φ* is the phase. If we want to generate a random surface, it is necessary that the phase *Φ* ∈ [0,π] or *Φ* ∈ [−π/2,π/2] according to a uniform distribution, so that cos(*Φ*) ∈ [−1,1]. It's remarkable that, as consequence of the relation cos(*π* − *Φ*) = −cos(*θ*), the spreading interval of *Φ* can't be longer than *π*, otherwise problems of uniqueness would arise.

In order to decrease the computational cost, we will take the oscillation frequencies v_x and v_y as integers: $v_x = m$ *and* $v_y = n$, *(m, n* $\epsilon\, Z$). This allows us to rewrite equation (1) as:

$$\cos\left(\vec{k}_{mn} * \vec{r} + \phi\right) = \cos\left(2\pi(mx + ny) + \phi\right);\, \vec{k}_{mn} = 2\pi(m,n) \qquad (3)$$

If we let *m,n* to take positive and negative values with the same probability, we could create a surface without a preferred oscillation direction. For the sake of simplicity, we will restrict the spatial frequencies introducing a cutoff frequency for the higher frequencies and another for the lower ones. The cutoff for higher frequencies is given by:

$$v_x^{max} = M;\, v_y^{max} = N \qquad (4)$$

As spatial frequencies take negative values, the cutoff for lower frequencies is given by:

$$v_x^{min} = -M;\, v_y^{min} = -N \qquad (5)$$

Thus, the wavelength is given by

$$\lambda_x^{min} = \frac{1}{M};\, \lambda_y^{min} = \frac{1}{N} \qquad (6)$$

The amplitude of the waves can be modulated to represent different physical phenomena. In this way the surface equation *f*(*x,y*) will be sum of different wave functions and can be expressed as:

$$f(x,y) = \sum_{m,n} A_{m,n} \cos\left(\vec{k}_{mn} * \vec{r} + \phi\right) \qquad (7)$$

If we let the A_{mn} coefficients to obey a Gaussian distribution, $f(x,y)$ will be a valid surface from a mathematical point of view, but will lack physical reality as it won't have a "natural" aspect. This is due to that the simulated coatings for the lower frequency oscillations have an amplitude greater than the amplitude for the higher frequency oscillations. Thus, in the discrete case, amplitudes will obey a distribution given by:

$$A_{m,n} = a(m,n) \sim h(m,n) = \frac{1}{(m^2 + n^2)^{\beta/2}} \qquad (8)$$

where β is the spectral exponent which tells how fast the higher frequencies are attenuated.

According to reference [2], spectral exponent is related to fractal dimension of the surface if the Fourier series has infinite terms. As we have finite frequencies, $a(m,n)$ will be given by reference [1]: $a(m,n) = g(m,n)h(m,n)$, where $g(m,n)$ will be a random function obeying a Gaussian distribution that allows us to have different random variations without limit.

On the other hand, phase Φ will be obtained from a random function $u(m,n)$, which obeys a normal distribution in intervals $[-\pi/2, \pi/2]$ or $[0, \pi]$:

$$\phi(m,n) = u(m,n) \qquad (9)$$

To create a 2D random surface, $f(x,y)$ will be a double sum in frequencies:

$$f(x,y) = \sum_{m=-M}^{M} \sum_{n=-N}^{N} a(m,n) \cos\left(2\pi(mx + ny)\right) + \phi(m,n) \qquad (10)$$

where x and y are the spatial coordinates; m and n are the spatial frequencies of x and y, respectively; $a(m,n)$ is the amplitude coefficient and $\Phi(m,n)$ is the phase.

This last expression clearly shows that $f(x,y)$ is periodic. To generate a surface without an obvious periodicity to better represent the coating, we need to cutoff $f(x,y)$ in such a way that the new surface has the previous properties. It's as easy as to restrict the spam of x and y values. Boundary values of the new surface are given by the total periodicity of $f(x,y)$, which is determined by the slower oscillations ($m = n = 1$).

The spatial frequencies with $m = n = 1$ give rise to a period length of 1 in every direction. So, if we want to build a rectangular ($a \times b$) random surface, it can be done in the domain $[a, a+1] \times [b, b+1]$ or in smaller ones.

Taking into account the questions mentioned above and in order to generate random circumferences in Comsol Multyphisics,[2] it is required to define some parameters as the maximum spatial frequency (N); spectral exponent (b) and the medium radius of the circumference (i.e., to define the pellets size) (r1). For simplicity we take $N = M$. Next, to generate the amplitudes we need a random function that obeys a Gaussian distribution whose standard deviation is the deviation from r1. To create the phase Φ we generate a random function that obeys an uniform distribution in $[0,\pi]$. Then, to build the circumference, we use a “Parametric Curve” node incorporating all the aforementioned properties. Finally, to introduce the Fourier series we have used the *sum* command of Comsol Multyphisics:

$$x = r1\cos(2\pi s)(1 + 0.1sum(if((m! = 0),\left((m^2)^{-b/2}\right)g1(m)\cos(2\pi ms + u1(m)),0),m, -N,N)) \quad (11)$$

$$y = r1\sin(2\pi s)(1 + 0.1sum(if((m! = 0),\left((m^2)^{-b/2}\right)g1(m)\cos(2\pi ms + u1(m)),0),m, -N,N)) \quad (12)$$

Supporting Information S3. XPS analysis of Al_2O_3/PZT samples

Figure S3 illustrates the XPS survey spectrum taken for a set of Al_2O_3/PZT pellets. The table gathers the atomic percentages of each element. The analysis of the surface of the pellets indicates that, although Al and O are the most abundant elements, signals due to PZT are also detected. This analysis suggests that, although the coverage of the pellet surface is almost complete, small zones of PZT are not fully covered by alumina. Similar evidence could be inferred for Ru-Al_2O_3/PZT pellets.

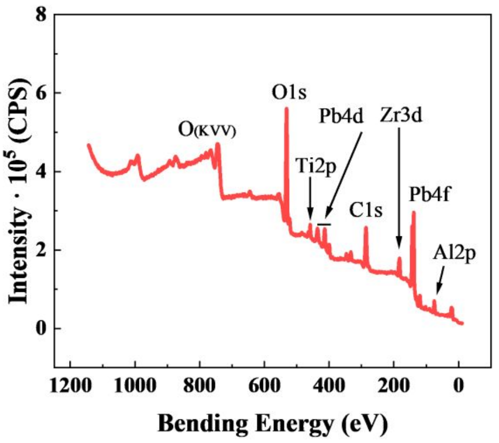

	Al	O	C	N
%at	18.8	39.4	29.3	3.2
	Pb	Zr	Ti	
%at	4.5	2.9	1.9	

Figure S3. XPS survey spectrum of a set of Al_2O_3/PZT pellets.

Supporting Information S4. XPS analysis and TEM micrographs of Al_2O_3 and Ru-Al_2O_3 powder samples.

Figure S4.1 (a) shows the XPS survey spectra of (a) Al_2O_3 and (b) Ru-Al_2O_3 powder samples. In both cases, signals associated to Al (Al2p, Al2s), O (O1s) and C (C1s) can be observed. The signal marked with C1s in the Ru-Al_2O_3 sample overlaps with that of Ru3d photoelectrons, the most intense photoelectron peak of this element. Despite the overlapping of the Ru3d and C1s peaks, the concentration of ruthenium can be estimated in 0.9% from the analysis of the less intense Ru3p signal. This is a reasonable value considering the agglomeration and partial distribution of the ruthenium (2% wt. added) in the Al_2O_3 powder internal pores. According to the position of the Ru3p3/2 signal (462 eV), it is expected that Ru nanoparticles are in an oxidized form, as expected from the calcination treatment in air of the Ru containing precursor powder.

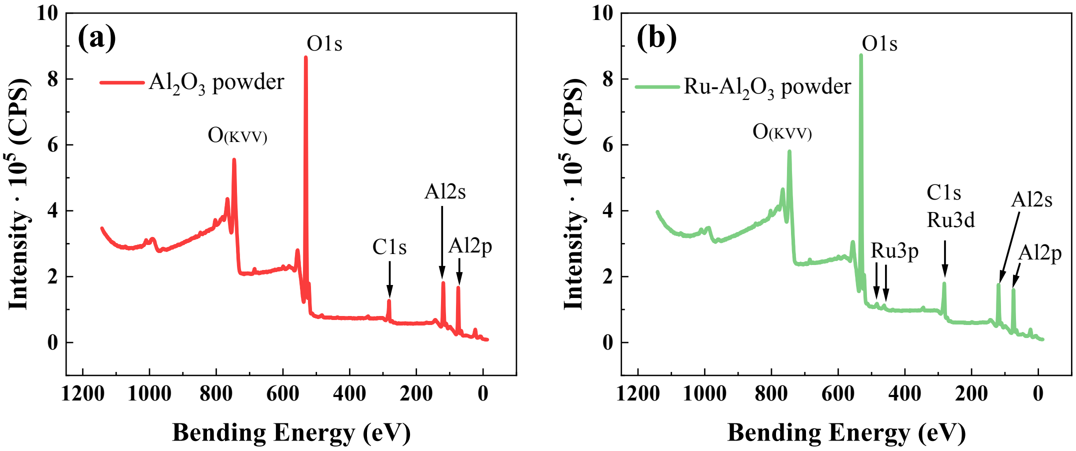

Figure S4.1. XPS survey spectra of Al_2O_3 (a) and Ru-Al_2O_3 (b) powder samples.

Figure S4.2 shows different TEM micrographs of the Al_2O_3 and Ru-Al_2O_3 powders. Based on those micrographs we estimate the mean size of aggregated Ru-particles (120 ± 6 nm). In addition, the figure also includes the EDX spectra to corroborate the presence of Ru nanoparticles in the case of the Ru-Al_2O_3 powders.

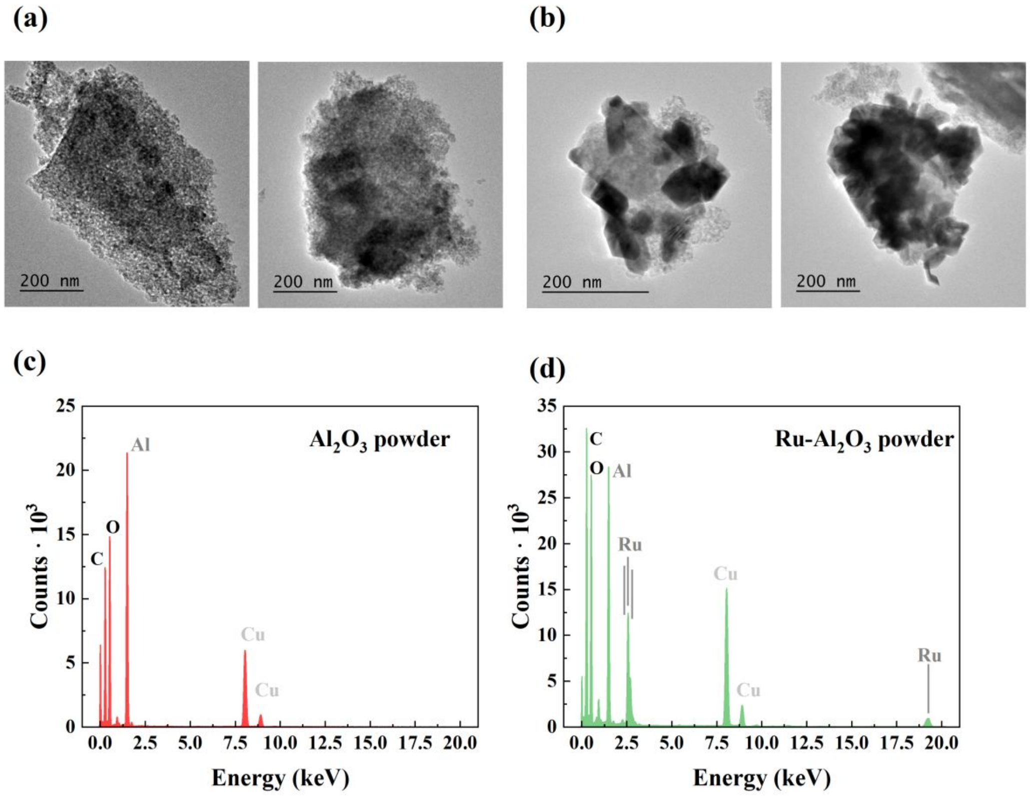

Figure S4.2. (a – b) TEM micrographs and (c – d) EDX spectra of Al_2O_3 and Ru- Al_2O_3 powder samples, respectively. The Cu signals in the EDX spectra are due to the sample holder.

Supporting Information S5. Lissajous plots at different applied voltages.

Figure S5 shows the Lissajous plot for the PZT configuration for increasing values of the applied voltage. As observed, the slope of the right and left side-lines in the plot increases (m3>m2>m1) with the applied voltage. This behaviour suggests that at the lower voltages the plasma does not fully occupy the inter-pellets space but expands at higher voltages. Similar behaviour is observed for the Al_2O3/PZT and Ru-Al_2O_3/PZT configurations.

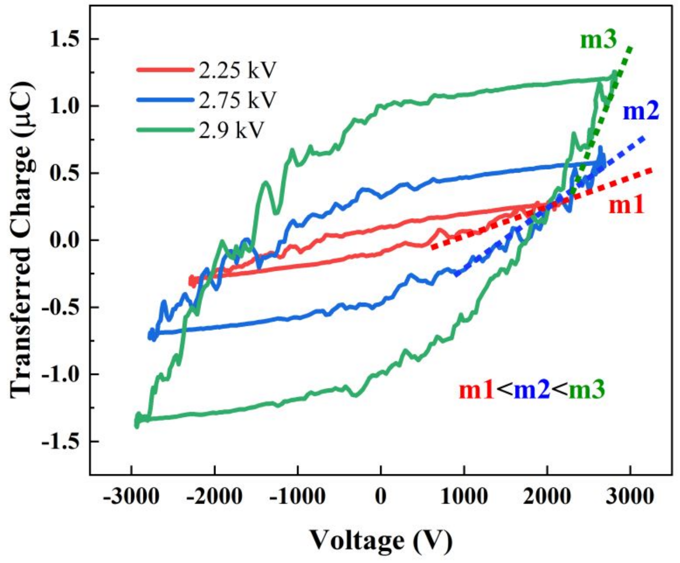

Figure S5. Lissajous plots for the PZT configuration at different applied voltages. Operating conditions: 5 mm PZT barrier, 5 kHz frequency, ambient temperature.

Supporting Information S6. Evolution of the consumed power vs. the frequency (2.5 kV, ambient temperature).

Figure 6 in the main text shows the evolution of the reaction yield and energy efficiency as a function of the operating frequency (1-5 kHz) for a constant applied voltage of 2.5 kV at ambient temperature. Figure S6 depicts the consumed power for each one of these experiments.

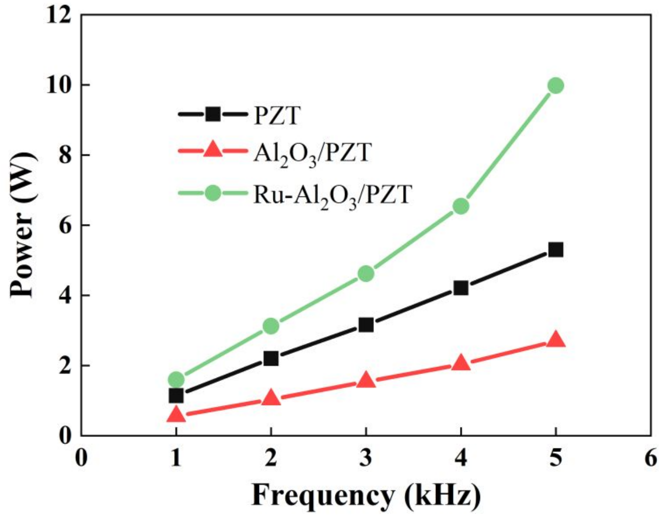

Figure S6. Average consumed power as a function of the frequency for an applied voltage of 2.5 kV at ambient temperature.

Supporting Information S7. Evolution of the consumed power vs. the frequency (2.5 kV, 190 °C).

The study at 190ºC was carried out for a fixed voltage of 2.5 kV and varying the frequency in the range of 1-3 kHz (see Figure 4(b) and (d) in the main text). Figure S7 shows the evolution of the consumed power of the reactor working under these conditions. As observed, for the three different configurations (working at 190ºC) the consumed power tends to similar values for frequencies ≤ 2.5 KHz, with slightly lower values for the Al2O3/PZT configuration.

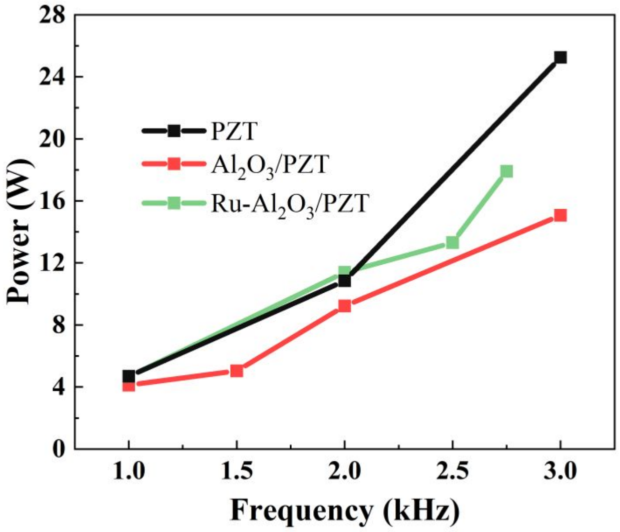

Figure S7. (a) Average consumed power as a function of the frequency for an applied voltage of 2.5 kV and a temperature of 190º.

REFERENCES

(1) Michael F Barnsley *et al.* The science of fractal images. Springer, 1988.

(2) Bjorn Sjodin. How to Generate Random Surfaces in COMSOL Multiphysics. https://www.comsol.com/blogs/how-to-generate-random-surfaces-in-comsol-multiphysics/
